# A machine learning-derived hypoxia- and lactylation-associated gene signature for prognostic stratification and immune landscape characterization in lung adenocarcinoma

**DOI:** 10.3389/fimmu.2026.1720885

**Published:** 2026-05-01

**Authors:** Guannan Wang, Yanan Wang, Hongfeng Wu, Hua Huang, Xuanguang Li, Wenhao Zhao, Yongwen Li, Hongyu Liu, Jun Chen

**Affiliations:** 1 Department of Lung Cancer Surgery, Center of Thoracic Surgery, Tianjin Medical University General Hospital, Tianjin, China; 2 Tianjin Key Laboratory of Lung Cancer Metastasis and Tumor Microenvironment, Tianjin Lung Cancer Institute, Tianjin Medical University General Hospital, Tianjin, China

**Keywords:** chemotherapy, histone lactylation, hypoxia, immunotherapy, lung adenocarcinoma

## Abstract

Lung cancer is one of the most frequently diagnosed cancers and the leading cause of cancer-related death worldwide. Hypoxia and histone lactylation are emerging regulators of tumor progression and immune escape. However, their concurrent enrichment and immune-associated features in lung adenocarcinoma (LUAD) remain unclear. In this study, we employed a combination of hypoxia- and lactylation-related genes to construct a new prognostic model using LASSO, XGBoost, and Random Forest algorithms. Single-cell RNA-seq (scRNA-seq) data were integrated to assess cell-type-specific expression. Functional enrichment, immune infiltration, TIDE-based immunotherapy response prediction and drug sensitivity analyses were performed to elucidate the biological characteristics of the subtypes and to explore their potential clinical and therapeutic implications. Promotion of lung cancer cell proliferation by the core gene polyadenylate-binding protein 1 (PABPC1) was verified by qRT-PCR, CCK-8, colony formation, wound healing and Transwell assays in two LUAD cell lines. In conclusion, we successfully established a new hypoxia- and lactylation-related gene-based model with value for predicting the prognosis of LUAD patients. By integrating multiple bioinformatic analyses and combining them with cell-based experimental validation, we found that cytoplasmic PABPC1 is a potential prognostic marker for lung cancer.

## 1 Introduction

As one of the most common malignant tumors, lung cancer is involved in approximately 2 million new cases and causes 1.76 million deaths each year, ranking first among cancer-related deaths (1). Although current diagnostic workflows integrate multiple imaging modalities with histopathological evaluation of biopsy specimens, these approaches still lack sufficient sensitivity for detecting early-stage disease (2). Over the last two decades, the identification of targetable oncogenic genomic alterations has fundamentally reshaped the treatment paradigm for non-small cell lung cancer (NSCLC) across both early and advanced settings, and newly discovered molecular drivers continue to emerge as promising therapeutic targets (3). Nonetheless, there is an ongoing need to identify novel biomarkers and effective therapeutic targets is urgently needed to facilitate earlier detection and improve clinical outcomes.

Mounting studies have confirmed that the tumor microenvironment (TME) promotes cancer progression in many aspects. Hypoxia is a prominent feature of the TME of most solid tumors (4), including lung adenocarcinomas, and is caused by an imbalance between rapid cancer cell proliferation and inadequate oxygen supply due to vascular dysfunction (5). The presence of hypoxic regions is one of the independent prognostic factors for human cancer (6). Under hypoxic stress, the transcription factor HIF1-α initiates the hypoxic response and plays a key role in driving tumor invasiveness by promoting abnormal proliferation, metastasis, angiogenesis, and drug resistance (7). At the same time, hypoxia-induced increases in glycolytic flux promote cell growth during hypoxic injury (8). In LUAD, hypoxia-induced metabolic reprogramming and genetic alterations enable cancer cells to adapt to hypoxic environments and maintain proliferation under hypoxic conditions (9). Other studies have shown that hypoxia promotes tumor immune escape through multiple pathways by stabilizing and activating HIF-1α/HIF-2α, including enhancing the PD-1/PD-L1 axis and inducing the shedding of molecules such as MICA to evade immune surveillance (10). These hypoxia-induced adaptations are tightly coupled to the accumulation of lactate, which has emerged not only as a metabolic byproduct but also as a critical regulator of tumor progression and immune suppression.

The conventional view of lactate as merely a metabolic byproduct has been fundamentally transformed by recent discoveries revealing its critical role as a signaling molecule, epigenetic modifier, and key metabolic substrate in cancer (11). Some clinical studies have shown that high lactate levels correlate with poor outcomes across multiple tumors—including lymphoma, pancreatic, lung, prostate, and bladder cancers (12). Lactic acid efflux induces intracellular alkalinity, which accelerates glycolysis while acidifying the tumor microenvironment. The resulting low pH further promotes tumor proliferation and invasion (13). In lung adenocarcinoma, hypoxia-driven glycolysis leads to a significant accumulation of lactate, which not only acts as an alternative energy source for tumor cells, but also creates an immunosuppressive microenvironment by inhibiting immune cell function (14). It was also shown that, genetic or pharmacological inhibition of the lactate transporter MCT1 reduces lactate efflux and accumulation in lung cancer cells (15). Beyond metabolism, lactate mediates post-translational modifications—most notably histone lactylation—that reprogram gene expression and promote malignant phenotypes (16). Zhang C et al. demonstrated that elevated levels of total lysine lactylation and histone H3 lysine 18 lactylation (H3K18la) in NSCLC tumors are correlated with poor patient prognosis (17). Notably, lactate induces M2 polarization in macrophages, suppresses IFN-γ secretion by CD8^+^ T/NK cells, and reduces T-cell migration. Concurrently, it upregulates PD-1 on Tregs and provides metabolic support. Collectively, these alterations promote immune escape and limit the efficacy of immunotherapy (18). Against this background, the inhibition of lactate transport and lactate signaling pathways is emerging as a new therapeutic direction for lung cancer. Among the candidates developed to date, the MCT1 inhibitor AZD3965 has entered Phase I/II clinical trials (NCT01791595) as the first “lactate-targeted” candidate, highlighting the feasibility and clinical potential of intervening in lactate signaling (19). Hypoxia transcriptionally augments glycolysis and lactate export while enabling lactate re-uptake/signaling; accumulated lactate acidifies the TME and produces histone lactylation (e.g., H3K18la) to reprogram transcription, and can stabilize HIF via pseudo-hypoxia, forming a positive-feedback “hypoxia→lactate→more intense hypoxic phenotype” loop.

In LUAD, prognostic models have been developed based solely on either lactate metabolism-related genes (LMRGs) or hypoxia-related genes (HRGs). However, given the significant heterogeneity of LUAD and the intricate crosstalk between hypoxia and lactate metabolic pathways, these single-factor models fail to comprehensively capture the disease characteristics. In this study, we systematically integrated expression data from hypoxia- and lactylation-associated genes (HALARGs) to construct a prognostic signature and to explore their relationship with the immune landscape of LUAD. By integrating multiple-algorithm (i.e., LASSO, XGBoost, and Random Forests) alongside nested cross-validation to screen robust features, we established a clinically transferable optimal prognostic model. This work enhances our understanding of the concurrent enrichment of LMRGs and HRGs on LUAD prognosis and TME remodeling, while also aiding the identification of novel therapeutic targets.

## 2 Results

### 2.1 Identification of genes associated with hypoxia and lactylation in lung adenocarcinoma

**Figure 1:**
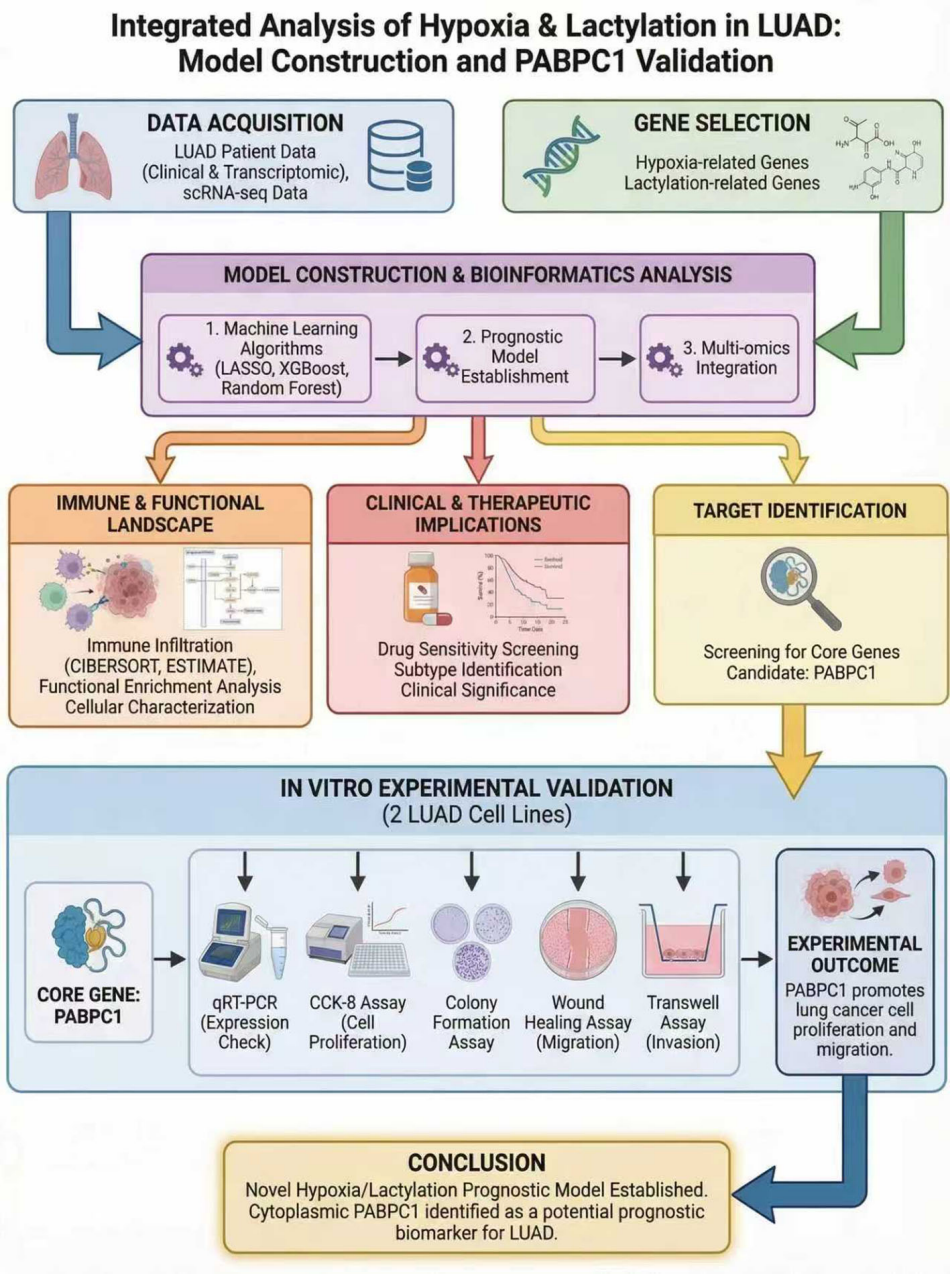
Schematic workflow of the study. The flowchart summarizes data collection, model construction, external validation, and downstream analyses.

**Figure 2:**
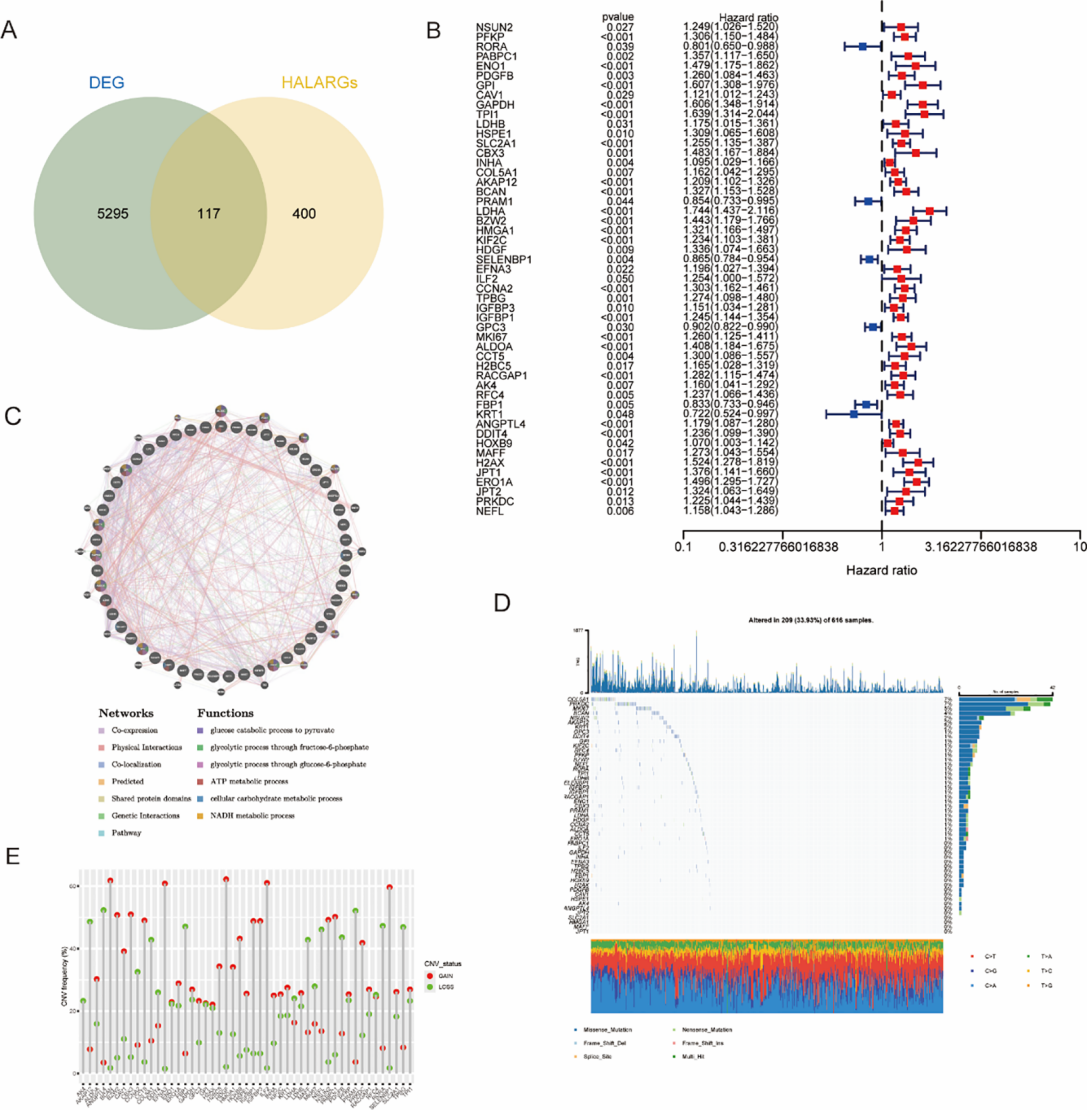
Identification of genes associated with hypoxia and lactylation in lung adenocarcinoma (LUAD). **(A)** A total of 117 overlapping genes were identified as hypoxia- and lactylation-related differentially expressed genes (DEGs). **(B)** Prognosis-related univariate Cox analysis. **(C)** Integration of biological networks of related prognostic genes analyzed by GeneMANIA. **(D, E)** Mutation frequency of DEGs in LUAD patients from TCGA cohort.

The overall study design and analytical workflow are illustrated in **Figure 1**. We obtained RNA sequencing and clinical data for 513 LUAD cases and 58 normal tissue samples from The Cancer Genome Atlas (TCGA) data portal (https://portal.gdc.cancer.gov/). Genotype-tissue expression (GTEx) data were also downloaded from the University of California, Santa Cruz (UCSC), Xena public resource platform (https://xenabrowser.net/datapages/). LUAD samples with missing survival time were excluded from this study. We used the R package “edgeR” to analyze genes in normal and tumor tissues in R (|log 2-fold change [FC]|>1 and P<0.05) and identified 5412 differentially expressed genes (DEGs). Then, an analysis to determine the genes overlapping between these DEGs and HALARGs was performed by the same method, and 117 of the DEGs were identified as also being HALARGs by Venn diagram visualization using the R package “VennDiagram” (**Figure 2A**). Fifty-one genes associated with the prognosis of hypoxic lactic acidification were further screened by univariate Cox analysis, and the results of the analysis of the related genes were presented in a forest plot (**Figure 2B**). In addition, the frequencies of copy number variations (CNVs) and somatic mutations in these 51 prognostic genes were determined, as summarized in **Figures 2D and E**.

Mutation analysis was then performed in 513 lung cancer tumor samples, with the mutation frequency of the 51 prognostic genes being 33.9%(**Figure 2D**), with COL5A1 having the highest mutation rate. In the CNV analysis, it was shown that BCAN, HDGF, EFNA3, and ILF2 exhibited high frequencies of copy number gain, while ANGPTL4 and PRAM1 had high frequencies of copy number loss, as shown in **Figure 2E**. In order to construct a related biological network, we further employed the GeneMANIA prediction server (http://www.genemania.org) to investigate the functional associations among these genes. The protein interaction network diagram in **Figure 2C** demonstrates that the core genes and their interactions are related to glycolytic pathway (glycolytic process) and energy metabolism (ATP/NADH metabolism), among others, reflecting the synergistic regulation of these genes/proteins via multiple biological relationships, such as co-expression, physical interaction, and co-localization.

### 2.2 Unsupervised clustering of HALARGs reveals prognostic subgroups with distinct functional features

**Figure 3:**
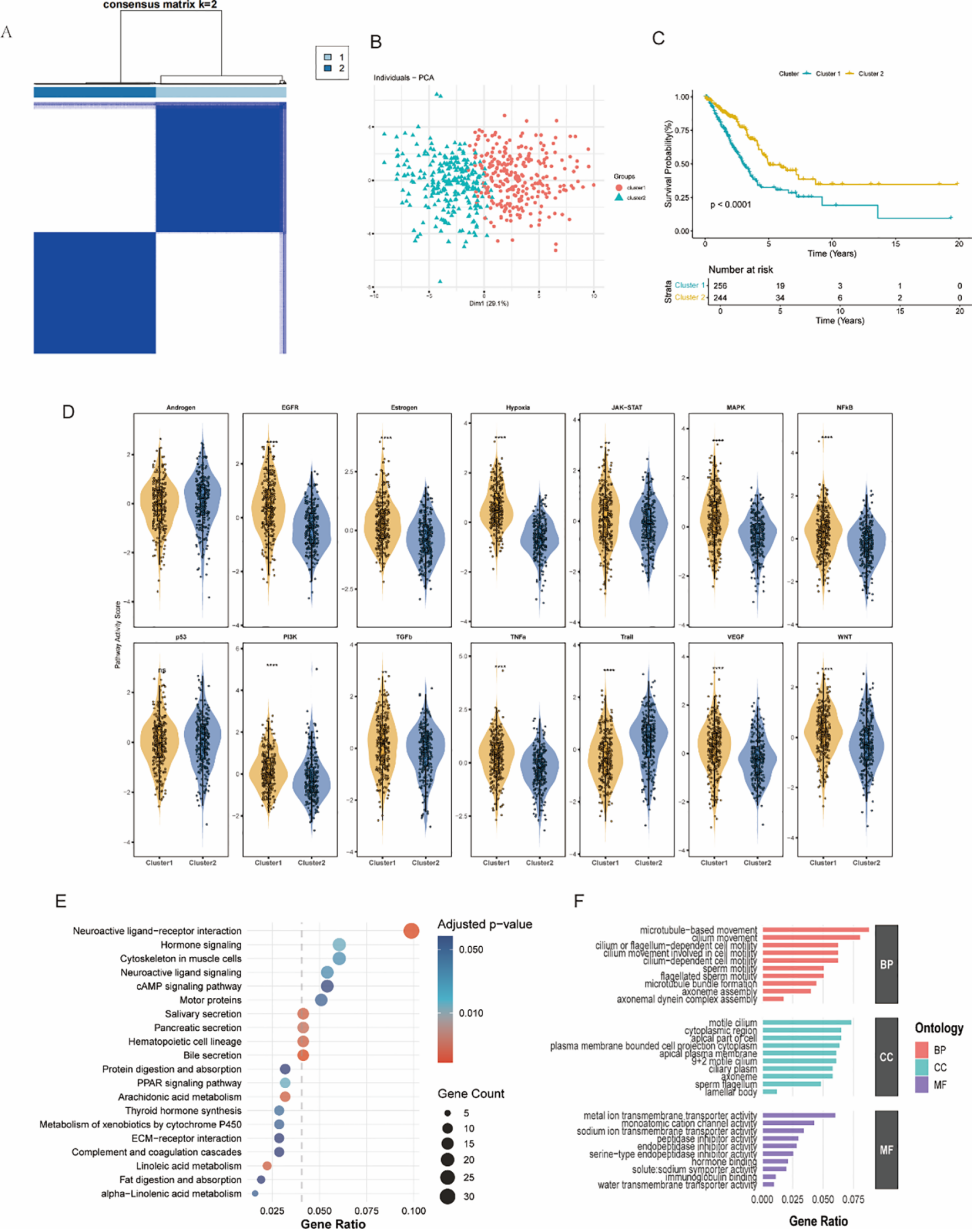
Identification of subgroups of HALARGs in LUAD. **(A)** Unsupervised consensus clustering analysis of LUAD. **(B)** PCA of C1 and C2 clusters. **(C)** Clinical survival analysis of C1 and C2 clusters. **(D)** Use of PROGENy to assess pathway activation in the two subtypes (ns *p* > 0.05; **p < 0.01; ****p < 0.0001). **(E)** KEGG enrichment analysis of the two subtypes. **(F)** GO enrichment analysis of the two subtypes.

Using unsupervised consensus clustering of 51 HALARGs, we classified LUAD patients (n=500) into two distinct molecular subtypes. The consensus matrix heatmap demonstrated stable clustering when k = 2 (**Figure 3A**), and principal component analysis (PCA) confirmed that the two clusters could be differentiated by their transcriptomes (**Figure 3B**). Survival analysis was performed using the “survival” R package (version3.5.7), and Kaplan–Meier survival analysis showed that the two clusters differed significantly in overall survival (OS), with the prognosis of Cluster 1 being significantly worse than that of Cluster 2 (P<0.001) (**Figure 3C**).

To explore the biological differences between the isoforms, we compared the pathway activity scores of Cluster 1 and Cluster 2 in 14 signature cancer-related signaling pathways. As shown in **Figure 3D**, Cluster 1 exhibited significantly higher activity in various oncogenic pathways, including EGFR, estrogen, JAK-STAT, MAPK, NFκB, TGF-β, TNF-α, VEGF, WNT, and hypoxia pathways, suggesting this cluster represents a more active and aggressive tumor subtype. In other pathways, the two subgroups were not particularly different. Cluster 2, on the other hand, exhibited higher PI3K pathway activity, reflecting its underlying biological differences. These results suggest different patterns of prognostic and other biological responses between the different subgroups.

Differential gene expression analysis was performed to identify DEGs between the two subtypes, followed by pathway enrichment analyses. KEGG pathway enrichment (**Figure 3E**) showed significant enrichment in neuroactive ligand–receptor interaction, hormone signaling, extracellular matrix (ECM)–receptor interaction, and motor protein-related pathways in the Cluster 1 subtype, highlighting potential roles of the genes activated in this subtype. Additionally, Gene Ontology (GO) enrichment analysis (**Figure 3F**) revealed that the DEGs upregulated in the Cluster 1 subtype were functionally enriched in biological processes related to microtubule-based movement, ciliary function, and axonemal assembly; cellular components such as plasma membrane and ciliary components; and molecular functions including metal ion transmembrane transporter activity and solute-sodium symporter activity. These pathways suggest that LUAD in patients in Cluster 1 has higher proliferative and metastatic potential.

### 2.3 Machine learning-assisted prognostic modeling

**Figure 4:**
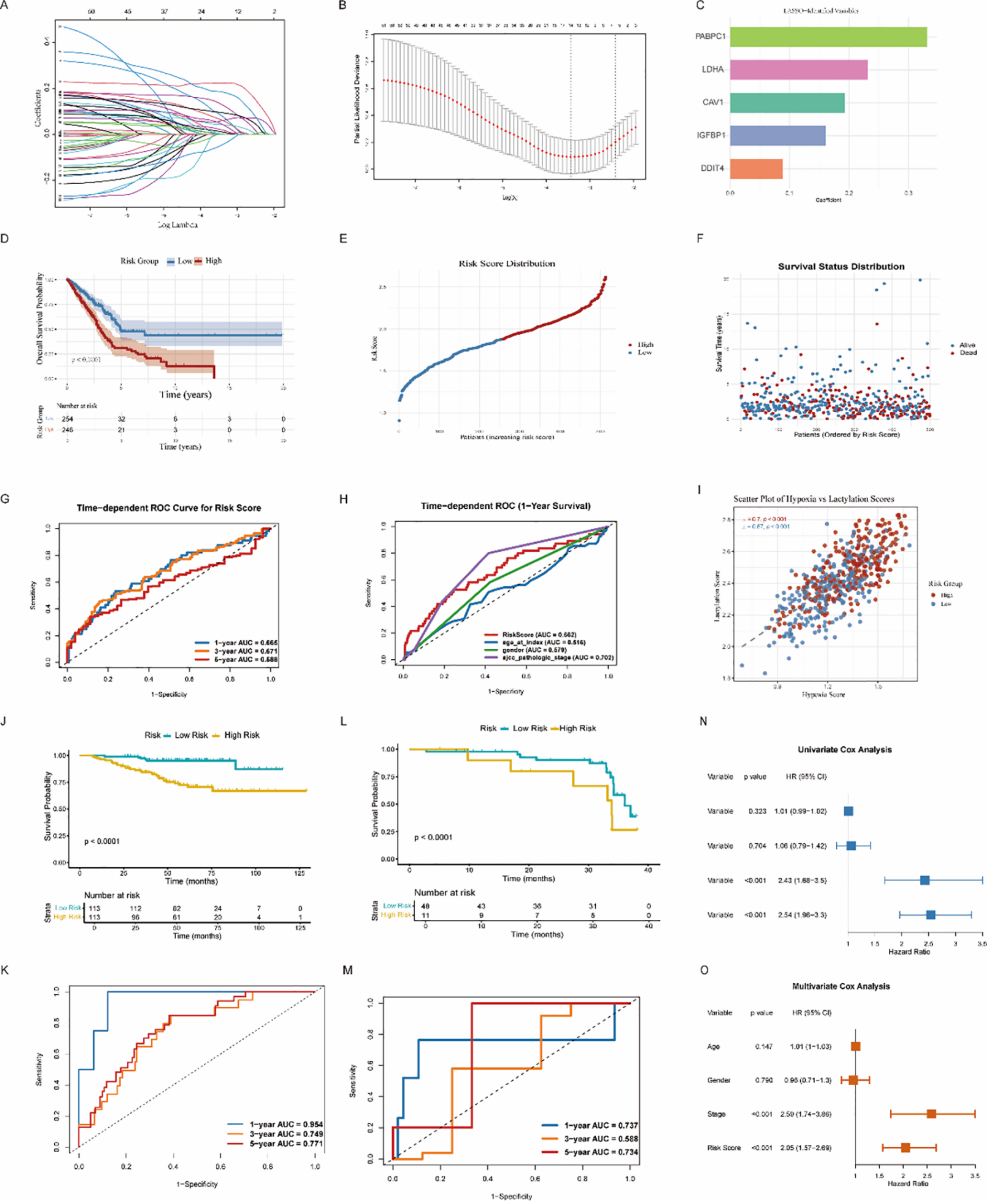
Construction of risk model. **(A, B)** LASSO regression analysis of prognostic genes after intergrated machine learning-based procedures to identify overlapping genes. **(C)** Bar graph of the coefficient index of the hub genes. **(D)**. Kaplan–Meier survival curves for overall survival (OS) are shown for both risk groups. **(E, F)** Risk score distribution and survival status in the two risk groups. **(G)** Time-dependent receiver-operating characteristic (ROC) curves of the risk score for predicting 1-, 3-, and 5-year OS. **(H)** Time-dependent ROC analysis indicating the predictive power of the risk signature and other clinical characteristics. **(I)** Scatter plot illustrating the correlation between hypoxia score and lactylation score in high- and low-risk groups. **(J, K)** Kaplan–Meier plots validating the predictive power of prognostic models with the GSE31210 dataset. **(L, M)** Kaplan–Meier plots validating the predictive power of prognostic models with the TMU dataset. **(N, O)** Forest plots of the univariate and multivariate Cox regression analyses.


To identify robust prognostic markers from the survival-associated HALARGs, we applied an integrated machine learning-assisted feature selection strategy using LASSO, XGBoost, and Random Forest. Specifically, we applied Least Absolute Shrinkage and Selection Operator (LASSO) regression, Random Forest, and Extreme Gradient Boosting (XGBoost) models—three widely used and complementary machine learning methods for feature selection in high-dimensional biological data. By determining the top-ranked genes that overlapped among all three models, we identified five candidate genes that consistently demonstrated strong prognostic relevance across the applied methods (**Supplementary Figure S1A**): PABPC1, LDHA, CAV1, IGFBP1, and DDIT4. This integrative approach ensured that the selected genes were not only statistically significant but also biologically robust and stably detected irrespective of the algorithm. These five genes were retained for further validation and downstream modeling. Expression levels and coefficients for each gene, derived using LASSO–Cox regression analysis, were then calculated and used to determine a risk score for a given sample (**Figures 4A–C**) via the following formula:

$$\begin{array}{c} Risk\;score\;=\;\beta_{P}ABPC1\times \;Expression_{PA}BPC1+\beta_{LDHA}\\ \times \;Expression_{LDHA}+\;\beta_{CAV1}\\ \times \;Expression_{CAV1}+\;\beta_{IGFBP1}\times \;Expression_{IGFBP1}+\;\beta_{DDIT4}\\ \times \;Expression_{DDIT4}, \end{array}$$

where β is= the coefficient of each gene. After calculating the median risk score, all of the samples of the training cohort were divided into high-risk and low-risk groups in this way. The survival curves show that the group with a high risk score has a worse prognosis (**Figure 4D**). **Figures 4E, F** show the corresponding risk score distribution and survival status. In the training cohort, the AUC values of risk score at 1, 3, and 5 years were 0.665, 0.671, and 0.588, respectively (**Figure 4G**). Moreover, the AUC (**Figure 4H**) values of risk score, age, sex and clinical stage were 0.662, 0.516, 0.579, and 0.702, respectively. These findings suggest that the risk model has value for predicting the prognosis of LUAD patients. To explore the relationship between tumor hypoxia and lactylation, we performed a joint scatterplot analysis based on hypoxia score and lactylation score for LUAD samples. As shown in **Figure 4I**, the two variables were positively correlated (Spearman’s r = 0.43, P < 0.001), indicating that hypoxia- and lactylation-related transcriptional states tended to co-occur in LUAD samples. Notably, samples categorized as “high-risk” according to the HALARG classification were predominantly clustered in the upper right quadrant of the scatterplot, suggesting that both scores were elevated simultaneously. This supports the possibility of functional synergy between lactylation and the hypoxic TME to promote LUAD progression.

To verify the reliability and validity of the prognostic model, we used transcriptomic sequencing data from GSE31210, GSE3141, GSE26939 and clinical samples from our center for validation in subsequent analyses. All samples were categorized into low-risk and high-risk groups based on the established prognostic models. Similarly, survival analysis showed that low-risk patients had better overall survival (OS) than high-risk ones (**Figures 4J, L**). In the GSE31210 test set, the AUC values at 1, 3, and 5 years are 0.954, 0.749, and 0.771, respectively (**Figure 4K**). In the clinical sample transcriptomic dataset, the AUC values for 1-, 3-, and 5-year survival were 0.737, 0.588, and 0.734, respectively (**Figure 4M**). Similar trends were also observed in GSE3141 and GSE26939(**Supplementary Figures S1D–G**). The receiver-operating characteristic (ROC) curves from four independent external validation datasets consistently demonstrated that this prognostic model exhibits favorable predictive value for LUAD patients. To further benchmark the prognostic performance of our signature against previously reported LUAD models, we performed a head-to-head comparison of time-dependent AUC values at 1, 3, and 5 years using the same evaluation framework. As shown in **Supplementary Figure S1H**, the HALARG model achieved AUCs of 0.67, 0.67, and 0.59 at 1, 3, and 5 years, respectively. Compared with the published hypoxia-based model, our HALARG model showed consistently higher AUC values across all three time points. Compared with the published lactylation-based model, the HALARG model showed a higher 3-year AUC, while the 1-year and 5-year AUCs were slightly lower. Overall, these results indicate that integrating hypoxia- and lactylation-associated features provides competitive and generally more balanced prognostic performance than single-process models, while also linking the risk signature to immune and single-cell characteristics in LUAD.

Both univariate and multivariate Cox regression analyses showed that risk score was an independent prognostic factor with high stability and predictive value in LUAD compared with other clinical characteristics (**Figures 4N, O**). Waterfall plots were also created to visualize the gene mutation profiles in the low- and high-risk groups (**Supplementary Figures S1A, B**). These profiles were generally similar, with genes such as TP53, TTN and MUC16 being the primary targets. However, the high-risk group had a slightly higher frequency of mutations in driver genes such as TP53, and its overall mutational burden was slightly higher than that of the low-risk group.

### 2.4 Immune cell infiltration in the risk groups

**Figure 5:**
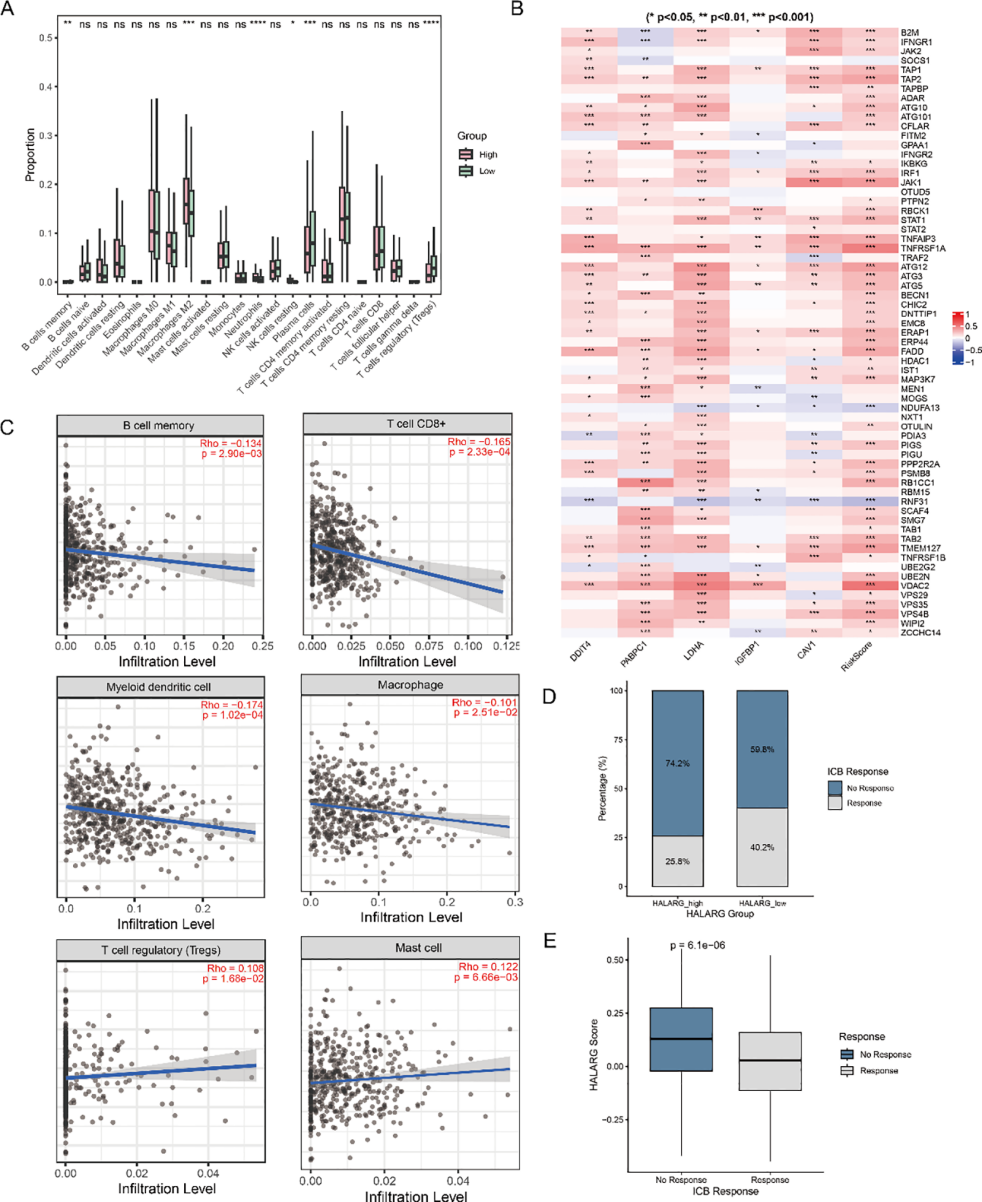
Analysis of immune cell infiltration and associations with the target genes. **(A)** CIBERSORT-estimated proportions of 22 immune cell types in high- and low-risk groups. **(B)** Heatmap of correlations between the hub genes and marker genes associated with escape of cells from immune surveillance (*p < 0.05; **p < 0.01; ***p < 0.001; ****p < 0.0001). **(C)** Correlation between the expression of target genes and the level of infiltration of CD8^+^ T cells, macrophages, and dendritic cells, among others, based on the TIMER database. **(D)** Proportions of predicted responders and non-responders in the HALARG-high and HALARG-low groups. **(E)** Comparison of HALARG scores between predicted responder and non-responder groups.

Since hypoxia and lactylation are closely related to the tumor immune microenvironment, we assessed immune cell infiltration in the high- and low-risk groups using CIBERSORT (**Figure 5A**). Compared with the low-risk group, the high-risk group exhibited higher total macrophage counts, but the increase was primarily derived from M2 macrophages. Moreover, dendritic cells (DCs) were predominantly quiescent/immature DCs rather than activated ones. Concurrently, CD8^+^ T-cell counts were lower in the high-risk group than in the low-risk group. These findings collectively indicate that the TME in the high-risk group presents an immune landscape characterized by myeloid bias and functional suppression, rather than an effective antitumor immune response. Linkage maps between the five characterized genes and different immune cells were plotted with the linkET package to further analyze the gene–cell interactions (**Supplementary Figure S1I**). The associations of the five selected genes with immune escape genes obtained from the literature were analyzed (**Figure 5B**). Correlation analysis of the single gene PABPC1 with different cell types was also performed with the online tool TIMER, and its expression level was found to be significantly correlated with a wide range of infiltrating immune cells (**Figure 5C**). Specifically, PABPC1 was significantly negatively correlated with the infiltration levels of antitumor immune effector cells such as memory B cells, CD8^+^ T cells, myeloid dendritic cells, and macrophages, while being significantly positively correlated with the infiltration levels of immunosuppressive cells such as regulatory T cells and mast cells. This suggests that PABPC1 may promote tumorigenesis and progression by shaping an immunosuppressive TME through weakening antitumor immune responses and enhancing immunosuppressive mechanisms. To further evaluate whether the HALARG-based risk signature was associated with potential responsiveness to immune checkpoint blockade, we performed TIDE analysis in the TCGA-LUAD cohort. Compared with the low-risk group, the high-risk group exhibited significantly higher TIDE scores, accompanied by increased T-cell exclusion or dysfunction signals, suggesting a greater likelihood of immune evasion and a lower probability of benefiting from ICI therapy (**Figures 5D, E**). In addition, the proportion of predicted responders was reduced in the high-risk group. These findings extend our immune infiltration results and indicate that the HALARG signature may have value not only in prognostic stratification but also in identifying LUAD patients with distinct immunotherapy response tendencies.

### 2.5 scRNA-seq analysis of signature genes

**Figure 6:**
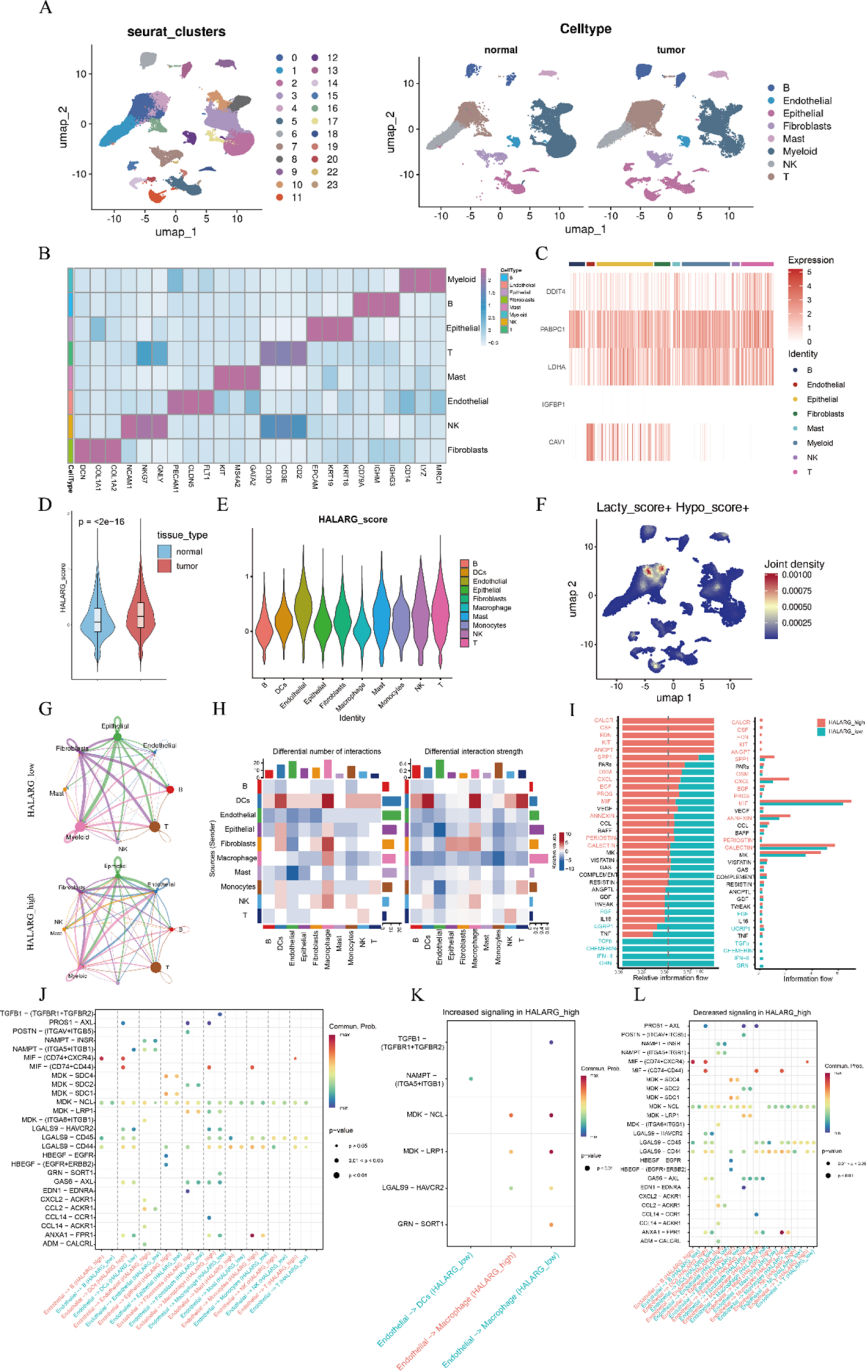
Single-cell transcriptomic profiling and intercellular communication of HALARG-related genes. **(A)** UMAP projection of cells from two tissue sources reveals 24 transcriptionally distinct clusters. UMAP projection colored by annotated major cell types and shown separately for normal and tumor samples. **(B)** Heatmap of representative marker genes validating cell type annotation. **(C)** Expression patterns of HALARG-related genes across cell types. **(D, E)** Violin plots showing elevated HALARG scores in tumor tissues and heterogeneous distributions across tumor-derived cell types. **(F)** Joint density map showing the spatial overlap of lactylation and hypoxia signals, highlighting cell subpopulations with concurrent enrichment of both programs. **(G)** CellChat analysis of communication networks in high- and low-HALARG-score groups. **(H, I)** Heatmap and bar plot illustrating differences in interaction numbers, strengths, and signaling information flow between groups. **(J–L)** Bubble plots depicting ligand–receptor pairs regulating communication between endothelial cells and other cells and summarizing up- or downregulated interactions in the high-HALARG-score group.

To comprehensively characterize the genes selected for incorporation into the model, we performed single-cell transcriptomic analysis using the publicly available dataset GSE131907. This dataset includes data on 11 tumor samples and 11 distant normal lung tissue samples, comprising a total of 88,142 cells that were mapped to a reference atlas and annotated into eight major cell types based on canonical marker genes (**Figures 6A, B**). Given the prognostic power demonstrated by our HALARG model, we further explored its characteristics at the single-cell level. We first assessed the expression of HALARGs across cell types, revealing differential expression patterns among various cell populations (**Figure 6C**). Using the “AddModuleScore” function in the Seurat R package (v5.0.1), we calculated HALARG scores for each cell based on model gene expression. As shown in **Figure 6D**, HALARG scores were significantly elevated in tumor tissues compared with those in distant normal tissues. To further investigate the role of HALARG-related genes within the TME, we extracted a total of 45,147 tumor-derived cells for in-depth analysis and grouped them into high and low HALARG score cohorts (**Supplementary Figure S2A**). Violin plots illustrated that endothelial cells exhibited the highest HALARG scores among all cell types (**Figure 6E**; **Supplementary Figure S3A**). To further delineate the single-cell distribution of hypoxia- and lactylation-related programs, we overlaid Lacty_score and Hypo_score onto the annotated UMAP atlas. Based on the cell-type map, the lactylation-related signal was mainly enriched in T cells, epithelial cells, and B cells, whereas the hypoxia-related signal was more prominently enriched in myeloid cells, fibroblasts, and epithelial cells. These findings indicate that the two programs were partially overlapping but not identical in their cellular distribution (**Supplementary Figure S3C**). Notably, joint density analysis showed that the concurrent enrichment of lactylation- and hypoxia-related signals was most evident in the T-cell region, suggesting that these two biological programs may co-occur in specific immune cell niches within the LUAD microenvironment (**Figure 6F**). In addition, representative feature plots of SLC2A1, LDHA, PABPC1 and ENO1 demonstrated heterogeneous yet partially overlapping expression patterns across the integrated atlas, further supporting the biological relevance of the HALARG signature (**Supplementary Figure S3D**). Using the median HALARG score as a threshold, we stratified tumor cells into high-risk group and low-risk group. To dissect the biological differences between these groups, we analyzed intercellular communication among the eight annotated cell types. Overall, the high-risk group exhibited an increased frequency of interaction and stronger signaling strength (**Supplementary Figure S2B**). In this group, endothelial cells, epithelial cells, and fibroblasts emerged as major communication hubs, displaying enhanced regulatory influence over immune cells and potentially contributing to an immunosuppr essive microenvironment. In contrast, the low-risk group showed a more balanced communication network, with more intense interactions between T cells and myeloid cells, suggesting a more activated immune state (**Figures 6G, H**). We further identified and prioritized key signaling pathways with significant differences between the groups. Cells in the high-risk group activated multiple pathways associated with tumor progression, including EGF, SPP1, CSF, KIT, and EDN (**Figure 6I**). Notably, endothelial and fibroblast cells in the high-risk group exhibited greater signal output, primarily through MIF, VEGF, and GALECTIN pathways, enhancing their regulatory influence on immune cells. By contrast, the low-risk group displayed a greater balance between signaling output and input, with fibroblasts and myeloid cells acting as key signal transmitters, indicating distinct remodeling of the communication landscape (**Supplementary Figures S2E, F**). Given that endothelial cells had the highest HALARG scores, we further analyzed their ligand–receptor-based communication with other cell populations. As illustrated in **Figure 6J**, endothelial cells in the high-risk group exhibited more active intercellular communication, particularly through the MIF signaling pathway, which was notably enhanced between endothelial and immune cells, indicating a potential immunoregulatory role. Finally, we identified differentially expressed ligand–receptor pairs between the groups. In the high-risk state, MDK–LRP1 and TGFB1–TGFBR interactions were significantly upregulated between endothelial cells and macrophages, suggesting that these pathways mediate key regulatory functions. In contrast, the majority of immune-related ligand–receptor interactions were downregulated, collectively indicating that the high-risk phenotype may be associated with an immunosuppressive microenvironment (**Figures 6K, L**).

We then refined T-cell subclusters using canonical markers into CD4^+^ T_Naive, CD4^+^ T_Th, CD4^+^ T_Treg, CD8^+^ T_eff and γδ T-cell populations (**Supplementary Figure S3B**). Compared with the HALARG-low group, the HALARG-high group showed a relative decrease in CD4^+^ naïve T cells and CD8^+^ effector T cells (**Supplementary Figure S3C**), whereas CD8^+^ T cells in the HALARG-high group exhibited significantly higher exhaustion scores (**Supplementary Figure S3D**), indicating an enrichment of exhausted CD8^+^ T cells in the high-HALARG microenvironment. For the myeloid compartment, cells were further annotated into cDC1, cDC2, cDC3, macrophages and monocytes (**Supplementary Figure S3E**). Although the overall proportion of macrophages was reduced in the HALARG-high group compared with the HALARG-low group (**Supplementary Figure S3F**), functional scoring revealed a clear shift in polarization: macrophages in the HALARG-high group preferentially displayed an M2-like program, whereas those in the HALARG-low group were biased toward an M1-like phenotype (**Supplementary Figure S3G, H**). Notably, these single-cell–level findings were broadly consistent with the bulk transcriptome–based analyses, supporting concordant immune features associated with HALARG-high versus HALARG-low states.

### 2.6 Knockdown of PABPC1 inhibits lung adenocarcinoma cell proliferation, migration, and invasion

**Figure 7:**
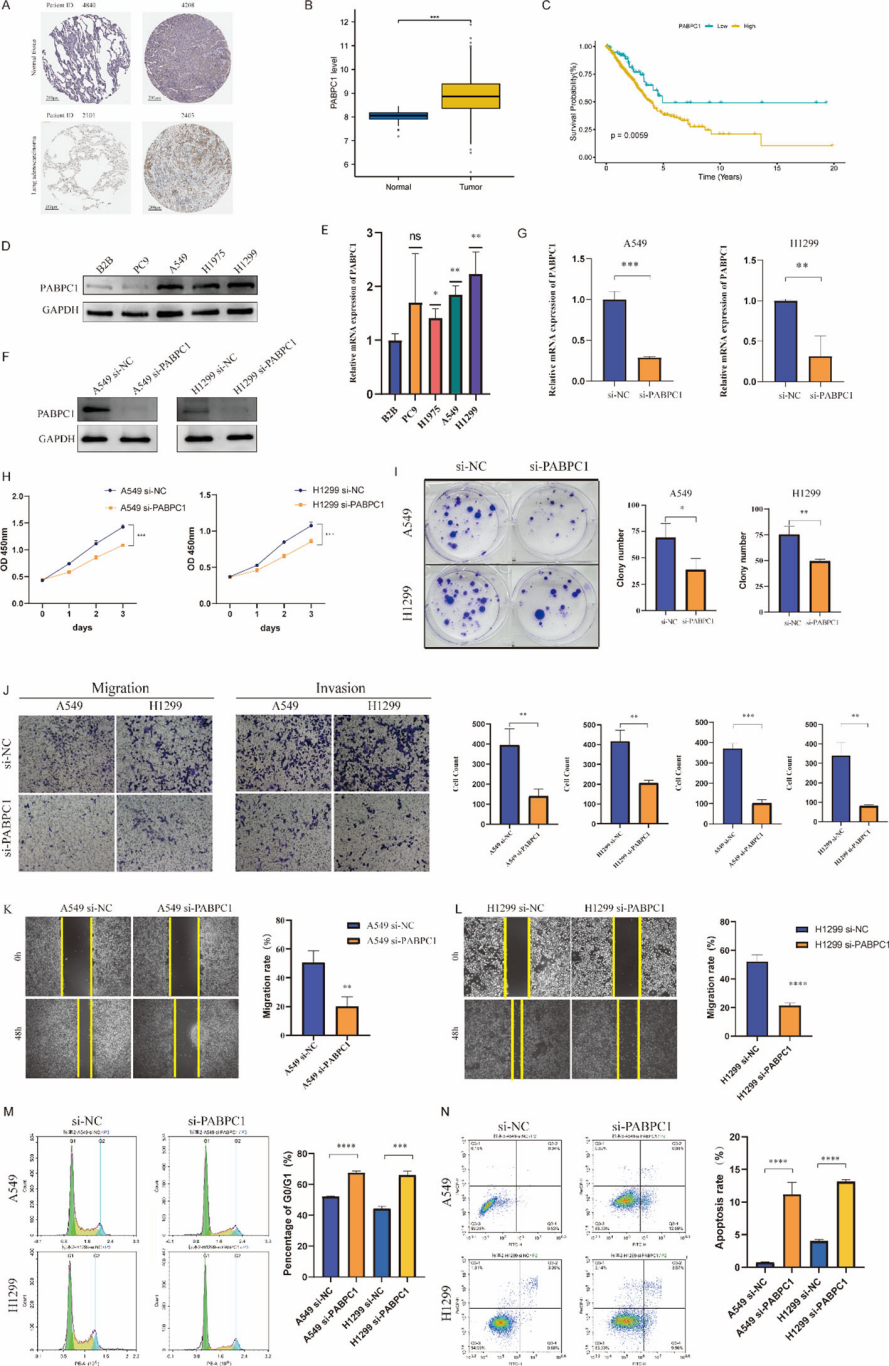
PABPC1 is highly expressed in LUAD and promotes cell proliferation and migration. **(A)** Representative immunohistochemistry images from the HPA database showing elevated PABPC1 protein expression in LUAD tissues. **(B)** TCGA and GTEx datasets confirming higher PABPC1 mRNA expression in LUAD samples compared with that in normal tissues. **(C)** Kaplan–Meier survival analysis indicating that high PABPC1 expression is associated with poorer prognosis in LUAD patients. **(D, E)** mRNA and protein expression of PABPC1 in lung cancer cell lines, with A549 and H1299 showing higher levels. **(F, G)** Establishment and validation of PABPC1-knockdown A549 and H1299 cell lines. **(H, I)** CCK-8 and colony formation assays demonstrating that silencing PABPC1 significantly inhibited LUAD cell proliferation. **(J)** Transwell assays showing that PABPC1 knockdown markedly reduced LUAD cell migration. **(K, L)** Wound-healing assays confirming that PABPC1-knockdown cells exhibited delayed scratch closure and wider gaps compared with siNC controls. **(M)** Flow cytometry analysis of cell cycle distribution revealing increased G_0_/G_1_ phase arrest in siPABPC1-treated A549 cells. **(N)** Flow cytometry analysis showing significantly increased apoptosis rates following PABPC1 knockdown. (*p < 0 . 05; **p < 0 . 01; ***p < 0 . 001; ****p < 0 . 0001).

Among the candidate genes derived from the HALARGs, PABPC1 was selected as the focus of further investigation. This decision was based not only on its significant prognostic value but also on it exhibiting the strongest correlation with immune cell infiltration, suggesting that it may play a crucial role in shaping an immunosuppressive TME. Immunohistochemistry results from the HPA database (https://www.proteinatlas.org/) revealed that PABPC1 protein expression was markedly elevated in LUAD tissues (**Figure 7A**). Consistent with this, TCGA and GTEx datasets further confirmed the upregulation of PABPC1 in LUAD tumor samples (**Figure 7B**). Survival analysis demonstrated that patients with high PABPC1 expression exhibited significantly worse prognosis (**Figure 7C**).

At both the protein and mRNA levels, we further validated that PABPC1 was highly expressed across lung cancer cell lines, with particularly elevated expression in A549 and H1299 cells (**Figures 7D, E**). Based on these findings, we established PABPC1-knockdown A549 and H1299 cell lines to further explore the role of PABPC1 in LUAD cell growth and migration (**Figures 7F, G**). Results from CCK-8 and colony formation assays showed that silencing PABPC1 significantly suppressed the proliferative capacity of both cell lines (**Figures 7H, I**). To further assess the role ofPABPC1 in migration, we performed Transwell assays, which revealed that PABPC1 knockdown markedly impaired the migratory ability of LUAD cells (**Figure 7J**). Consistent with this, wound-healing assays demonstrated that, compared with the control group (siNC), cells with PABPC1 knockdown exhibited significantly delayed wound closure and wider intercellular gaps (**Figures 7K, L**).

Moreover, we evaluated the effects of PABPC1 silencing on cell cycle progression and apoptosis using flow cytometry. In A549 cells, knockdown of PABPC1 led to a significant increase in the proportion of cells arrested at the G_0_/G_1_ phase (**Figure 7M**), along with a marked elevation in apoptosis rates (**Figure 7N**). Collectively, these results indicate that PABPC1 plays an important role in promoting LUAD cell proliferation and migration. Its silencing not only induces G_0_/G_1_ cell cycle arrest, but also significantly enhances apoptosis, highlighting PABPC1 as a potential therapeutic target in LUAD. Together with the hypothesis-generating molecular docking results (**Supplementary Figure S4**), our findings suggest that PABPC1 warrants further exploration as a potential therapeutic vulnerability in LUAD.

## 3 Discussion

Lung adenocarcinoma (LUAD) is characterized by profound intratumoral heterogeneity and a dynamically evolving TME. In this study, we constructed a prognostic model based on hypoxia- and lactylation-associated genes (HALARGs), validated its performance across independent cohorts, and further linked it to immune infiltration and single-cell transcriptional programs. Our findings suggest that hypoxia and lactylation may potentially influence the remodeling of the microenvironment in LUAD, although the underlying mechanisms require further experimental validation.

Hypoxia profoundly influences cancer biology by shaping metabolic adaptation, invasive behavior, and therapy resistance through transcriptional and post-translational programs dominated by HIF-1α signaling (20, 21). Lactate, the major glycolytic metabolite, not only supports tumor metabolism but also acts as a signaling molecule that promotes macrophage polarization, immune suppression, and tissue remodeling (22). In this study, we systematically explored the roles of hypoxia and lactylation in LUAD prognosis using mRNA expression profiles and clinical data from the TCGA and GEO databases. Based on 51 HALARGs, two clusters, C1 and C2, were identified by unsupervised consensus clustering, and survival analysis showed that C1, characterized by higher HALARG expression, was associated with poorer prognosis. PROGENy analysis further revealed activation of multiple oncogenic pathways in C1, including hypoxia-related pathways. GO and KEGG enrichment analyses of differentially expressed genes (DEGs) between the two groups showed significant enrichment in the PPAR signaling pathway, arachidonic acid metabolism, and ECM–receptor interaction. Previous studies have shown that PPARγ is involved in lactate metabolism in multiple cancers and that its transcriptional activity can be altered by lactylation (23). In addition, arachidonic acid metabolites, including prostaglandins and leukotrienes, are important mediators of inflammation and cancer progression (24), and hypoxia can further enhance arachidonic acid metabolism to promote tumor-associated inflammatory responses (25).

To more accurately assess the prognostic value of these HALARGs, we combined multiple machine learning algorithms, namely, LASSO, XGBoost, and Random Forest, to screen the key genes, taking into account the model heterogeneity and improving the robustness of the screening results (26). A prognostic risk model was also constructed based on the expression profiles of the HALARGs identified by the integrated machine learning approach. Validation results from four additional independent GEO datasets were also used to show the stability of the model’s predictive performance. AJCC stage remains a readily available and strong prognostic factor. Our transcriptome-based score is intended to complement, not replace, staging by further stratifying risk and providing biological context. Larger prospective studies are needed to confirm its added clinical value.

The TME in LUAD is complex and dynamically evolving (27). As the tumor progresses, angiogenesis and metabolic changes lead to hypoxia within the tumor microenvironment, which further reshapes it (28). Compared with the low-risk group, the high-risk group exhibited an immunosuppressive immune landscape characterized by increased macrophage infiltration predominantly driven by M2 macrophages, reduced CD8^+^ T-cell infiltration, and impaired antitumor immune activity. CD8^+^ T cells are particularly vulnerable to hypoxia, which can accelerate terminal exhaustion and impair antitumor immunity (29). Although T cells may mount adaptive responses through HIF-1α-mediated programs, these mechanisms are often insufficient to restore effective immune activity in the high-risk setting (30). In addition, endothelial cells and fibroblasts in the HALARG-high group showed enhanced immunoregulatory signaling, including MIF, VEGF, and TGF-β-related pathways, collectively supporting a more suppressive microenvironment. TIDE analysis further showed that the high-risk group had higher TIDE scores and a lower predicted likelihood of response to immune checkpoint blockade, further indicating that the HALARG signature may capture differences in immune evasion and immunotherapy sensitivity. In contrast, the HALARG-low group exhibited more balanced intercellular interactions, accompanied by active T-cell/myeloid-cell crosstalk, suggesting a more immune-responsive state.

Finally, we analyzed the role of the gene PABPC1 in LUAD. PABPC1 encodes an RNA-binding protein that shuttles between the nucleus and cytoplasm and binds the 3′ poly(A) tail of eukaryotic mRNA (31). Increasing evidence indicates that PABPC1 is aberrantly expressed in a variety of cancers, including lung, gastric, breast, liver, and esophageal cancers, and that it may have value as a biomarker for future tumor diagnosis, therapy, and clinical applications (32). Researchers have also recently discovered that the makorin ring finger protein 3 (MKRN3)–PABPC1 pathway plays an important role in the pathogenesis of lung cancer (33). In this study, we verified that PABPC1 expression is significantly elevated in LUAD tissues and cell lines and that patients with higher PABPC1 expression have shorter survival and worse prognosis. Functional assays further showed that PABPC1 knockdown inhibited LUAD cell proliferation, migration, and invasion. In addition, our hypothesis-generating in silico screening and docking analysis suggested that PABPC1 may have potential druggability, although further biochemical and pharmacological validation is required.

Although our results suggest a potential relationship between hypoxia and histone lactylation in LUAD, several limitations of this work need to be considered. First, this study is retrospective in nature and based mainly on public datasets (from TCGA and GEO), which may introduce selection bias. To overcome this limitation, we intend to collect more clinical samples for further validation to confirm the prognostic and therapeutic relevance of our model in LUAD. Although our model showed potential in predicting prognosis and immunotherapeutic response, the underlying mechanisms linking hypoxia- and lactylation-related gene expression and tumor mutational burden, immune cell infiltration, and therapeutic sensitivity are still not fully understood. Future studies are needed to elucidate these interactions at the molecular level. There is also a need for *in vitro* experiments focusing on hypoxic environments and those featuring high lactate metabolism, as well as experiments exploring more deeply the specific biological mechanisms by which PABPC1 promote the development of LUAD.

## 4 Materials and methods

### 4.1 Data acquisition

We downloaded transcriptomic RNA-seq data (normal samples: n=58, tumor samples: n=513), clinical data (cases: n=500), and mutation data for LUAD via TCGA (http://cancergenome.nih.gov/). We then converted the integrated IDs to official gene symbols and subjected the data to log2 processing. The external validation datasets GSE31210, GSE26939, and GSE3141 were also obtained from the GEO database (https://www.ncbi.nlm.nih.gov/geo/) and batch-normalized. A hypoxia-associated gene set was also extracted from the GSEA database, and a lactylation-associated gene set was based on 327 lactylation-associated genes compiled from previous studies (**Supplementary Table S1**) (34). A total of 33 LUAD patients from the Department of Lung Cancer Surgery at Tianjin Medical University General Hospital were also enrolled in this study. Their surgically resected tumor tissues and adjacent normal tissues were collected for transcriptomic sequencing. Clinical information, including age, sex, and TNM stage, was also obtained from electronic medical records. All patients provided written informed consent (Ethics Approval No.: IRB2019-KY153).

To explore the genomic alterations of differentially expressed HALARGs, we first analyzed the somatic mutation profiles of these genes using the “maftools” package in R. TCGA genomic data were evaluated for CNVs, including amplifications and deletions. In addition, gene–gene interaction and functional networks were constructed using the GeneMANIA prediction server (http://www.genemania.org).

### 4.2 Differential expression analysis and prognostic value

Differential expression analysis was performed using the DESeq2 package in R (version 1.32.0). Genes with a false discovery rate (FDR) < 0.05 and an absolute fold change of ≥ 1 were considered as DEGs. Pairwise correlations between identified DEGs were assessed by calculating Spearman’s correlation coefficient. Functional interaction networks were constructed and gene function predictions were made using the GeneMANIA prediction server (http://www.genemania.org) (35). In addition, univariate Cox proportional risk regression was applied to assess the prognostic significance of each DEG by combining gene expression levels with survival time and survival status using the “survival” package in R (version 3.8.3).

### 4.3 Molecular subtyping and functional enrichment analysis

Cluster analysis was performed using ConsensusClusterPlus (36) with aggregated PAM clustering using Euclidean distances; all processes were repeated 1000 times to ensure the stability of supervised clustering. The optimal number of clusters was determined using plots of the empirical cumulative distribution function and average within-group agreement. The distributions of the two clusters were demonstrated using principal component analysis (PCA). In addition, PROGENy (Pathway RespOnsive GENes for activity inference) (37) was used to assess the activation of different cell signaling pathways between the two subgroups. Instead of relying directly on simple enrichment of gene sets, this approach uses experimentally validated pathway response gene signatures to more accurately infer signaling pathway activity. GSEA was performed in the enriched MSigDB collection (h.all.v7.0.cymbols.gmt) to determine the differences in regulation of hypoxia and lactylation gene set signaling pathways between the two subgroups. p< 0.05 was considered statistically significant.

### 4.4 Construction and validation of prognostic models

After univariate Cox regression analysis to identify 51 survival-associated DEGs, LASSO regression, XGBoost and Random Forest were used as complementary feature-selection methods to prioritize hub genes for prognostic model construction. A risk score was subsequently computed for each tumor sample based on the normalized expression levels of the selected hub genes and their respective regression coefficients derived from the model. Tumor samples were categorized into high- and low-risk groups using the median risk score as a threshold. The reliability of the model was further validated using the expression matrices and clinical data from the external test set GSE31210, as well as from 33 LUAD patients treated at the Department of Lung Cancer Surgery, Tianjin Medical University General Hospital. OS was analyzed using the Kaplan–Meier method. ROC analysis and AUC calculations were performed at 1, 3, and 5 years using the “timeROC” R package. In addition, univariate and multivariate Cox analyses were performed to determine the independent prognostic significance of certain factors.

To compare the prognostic performance of our model with previously published LUAD gene signatures, we reimplemented literature-based prognostic models according to the genes and scoring formulas reported in the original publications. Using the same normalized expression matrix and survival data from the TCGA-LUAD cohort, a risk score was calculated for each sample for every model. Time-dependent ROC analysis was then performed using the timeROC R package to calculate 1-, 3-, and 5-year AUC values under a unified evaluation framework. The HALARG model was further compared with representative hypoxia-based, lactylation-based, and other published LUAD prognostic signatures to assess its relative predictive performance.

### 4.5 Analysis of immune landscape

We used CIBERSORT analysis to quantify and assess the relative abundance of 22 different subsets of immune cells, including B cells, T cells, and NK cells, in the high- and low-risk groups defined by the median value of the prognostic risk score, in order to compare and predict the immune cell infiltration between the two groups. Immune evasion genes (38) were collected from previous studies, and their expression was analyzed for immune function correlations with the pivotal genes identified in the model by ssGSEA. Correlations of the expression of single genes with different immune cells were analyzed online using Timer (http://timer.cistrome.org). To visualize the bidirectional correlations of the target genes with multiple variables, we generated correlation butterfly plots, and also performed correlation analyses using Spearman’s method, with visualization of the results using the “linkET” package.

To further estimate the potential response to immune checkpoint blockade (ICB), TIDE analysis was performed using the normalized TCGA-LUAD expression matrix with the TIDEpy package (version 1.3.8). For each sample, the overall TIDE score, T-cell dysfunction score, and T-cell exclusion score were calculated. Samples with TIDE < 0 were defined as predicted responders, whereas those with TIDE > 0 were defined as predicted non-responders. We then integrated the TIDE-predicted response status with the HALARG signature to evaluate the associations of HALARG groups and HALARG scores with ICB response. Differences in TIDE-related scores and HALARG scores between groups were assessed using the Wilcoxon rank-sum test, and the distribution of predicted responders and non-responders between HALARG-high and HALARG-low groups was compared by chi-square test or Fisher’s exact test, as appropriate. In addition, two independent immunotherapy-related datasets, GSE126044 and GSE135222, were used for external validation, in which HALARG scores were compared between responder and non-responder subgroups.

### 4.6 Acquisition and analysis of scRNA-seq data

The scRNA-seq dataset was obtained from the Gene Expression Omnibus (GEO) under accession number GSE131907. Raw count matrices were processed and subjected to rigorous quality control (QC) to exclude low-quality cells and doublets, based on mitochondrial gene percentage, detected gene number, and UMI counts. After this QC, 88,142 high-quality cells were retained for downstream analysis. Dimensionality reduction and unsupervised clustering were performed using the Seurat R package (version 5.0.1), and cell types were annotated according to canonical marker genes (39). To evaluate functional states, we applied the “AddModuleScore” function in the Seurat R package to compute risk gene–based scores for each single cell. Furthermore, to investigate differences in intercellular communication between high- and low-score groups, we employed the CellChat R package (version 1.6.1), which enabled the inference and comparison of ligand–receptor signaling networks across conditions. UMAP-based visualization of HALARG score, Hypo_score, Lacty_score, joint density of hypoxia and lactylation signals, and representative gene expression patterns was performed using the scCustomize package (version 2.1.2). These analyses were used to characterize the single-cell distribution and co-enrichment of hypoxia- and lactylation-related transcriptional programs in LUAD.

### 4.7 Lung adenocarcinoma cells and transfection

The human lung adenocarcinoma cell lines A549, H1975, H1299, and PC9 were obtained from ATCC and cultured in Dulbecco’s modified Eagle medium (DMEM, PM150210; Priscilla, China) containing 10% fetal bovine serum (FBS) (164210-50; Priscilla, China). All of the cells were cultured in a humidified incubator at 37 °C with 5% CO_2_. For transfection, Lipofectamine 2000 (Invitrogen, USA) was used to transfect PABPC1 siRNA or random control siRNA (Ribobio), in accordance with the manufacturer’s instructions. The siRNA sequences of PABPC1 were as follows: sense strand 5′-GCUCCUAAAUGAUCGCAAATT-3′ and antisense strand 5′-UUUGCGAUCAUUUAGGAGCTT-3′.

### 4.8 Western blotting

After protein samples were extracted and quantified, aliquots were separated by 10% SDS-polyacrylamide gel electrophoresis (SDS-PAGE). Proteins were transferred to polyvinylidene difluoride (PVDF) membranes (Millipore, Billerica, MA, USA) by a semi-dry transfer system. Membranes were closed by incubation with a solution containing 5% bovine serum albumin (BSA) in TBST at room temperature with gentle shaking for 1 h. Subsequently, the membranes were incubated overnight at 4 °C with primary antibodies: anti-GAPDH antibody (60004-1-lg, 1:1000; Proteintech) and anti-PABPC1 antibody (10970-1-AP, 1:1000; Proteintech). After washing, membranes were incubated with horseradish peroxidase (HRP)-labeled secondary antibody (1:5000; Thermo Fisher Scientific, Inc.) for 1 h at room temperature. Protein bands were colored using Pierce ECL substrate (Thermo Fisher Scientific, Inc.).

### 4.9 RNA extraction and RT-qPCR

Total RNA was extracted using TRIzol reagent (#15596026CN; Invitrogen, USA), in accordance with the manufacturer’s instructions. Complementary DNA (cDNA) was synthesized from mRNA using a reverse transcription kit (#RR037A; TaKaRa, Japan). Quantitative real-time PCR (qRT-PCR) was performed to measure cDNA expression levels, with GAPDH used as an internal control. The qPCR primers used were as follows: GAPDH forward primer 5′-AAGGTCGGGAGTCAACGGATT-3′, reverse primer 5′-CTCCTGGAAGATGGGTGATGG-3′; and PABPC1 forward primer 5′-CAGGCTCACCTCACTAACCAG-3′, reverse primer 5′-GGTAGGGGTTGATTACAGGGT-3′.

### 4.10 Cell proliferation assay

Cell proliferation was assessed using the CCK-8 assay. Briefly, cells were seeded into 96-well plates and cultured for 24, 48, and 72 h. At the indicated timepoints, 10 μL of CCK-8 reagent was added to each well, followed by incubation for 2 h at 37 °C. The absorbance at 450 nm was then measured using a microplate reader to determine cell viability.

### 4.11 Colony formation assay

Cells transfected with siNC and siRNA were inoculated in six-well plates at 1000 per well and cultured for 14 days. Then, the cell population was fixed with 4% paraformaldehyde for 20 min, stained with 0.1% crystal violet for 10 min, washed gently with phosphate-buffered saline (PBS), and then allowed to air-dry naturally and photographed. The number of cells in each population was quantitatively analyzed using ImageJ software.

### 4.12 Transwell assay

Migration and invasion experiments were performed using Transwell chambers (Corning, USA) with a pore size of 8 µm. Experiments were performed in 24-well plates. For the invasion experiments, the upper chamber was pre-coated with Matrigel (BD BioCoat), whereas the migration experiments were performed without Matrigel coating. A 200 μL serum-free cell suspension containing 1×10^4^ cells was inoculated into the upper chamber, while 500 μL of medium containing 10% FBS was added to the lower chamber. After 48 h of incubation in a wet incubator at 37 °C with 5% CO_2_, non-migrated cells on the membrane of the upper chamber were removed with a cotton swab. Cells on the lower surface that had migrated/invaded were washed with PBS, fixed with 4% formaldehyde for 20 min, and stained with 0.1% crystal violet for 10 min. After washing three times with PBS and being allowed to air-dry naturally, the cells were imaged using an inverted microscope (CKX53; Olympus) and quantitatively analyzed by ImageJ software.

### 4.13 Scratch wound-healing assay

Cells were inoculated into six-well plates and cultured until they reached confluence. A linear scratch wound was created using a sterile 200 μL pipette tip, followed by washing of the cells with PBS to remove debris. Subsequently, the cells were cultured in serum-free medium and wound healing was monitored at 0 and 48 h using an inverted microscope.

### 4.14 Cell apoptosis and cell cycle assay

Apoptosis was assessed using the Annexin V-FITC Apoptosis Detection Kit (BD Biosciences), in accordance with the manufacturer’s instructions. Cells treated with different concentrations of degradants were collected, rinsed with pre-cooled PBS, and resuspended in 100 μL of binding buffer containing 5 μL of Annexin V-FITC and 5 μL of propidium iodide (PI). After 30 min of incubation in the dark at room temperature, 400 μL of binding buffer was added and the samples were immediately analyzed by flow cytometry. Data were analyzed using FlowJo software (Tree Star).

For experiments to analyze the cell cycle, A549 and H1299 cells were inoculated in two six-well plates (2×10^5^ cells/well) and treated with degradants at different concentrations for 12 h. Cells were harvested by trypsinization, rinsed with PBS, and fixed overnight at −20 °C in 75% ice-cold ethanol. Fixed cells were washed twice with PBS and then treated with 500 μL of RNase A (0.2 mg/mL, Biotronik) for 30 min at 37 °C, followed by staining with 50 μg/mL PI (BD Biosciences) for 15 min in the dark. Cell cycle distribution was analyzed using a NovoCyte flow cytometer (Agilent Technologies).

### 4.15 Virtual screening and molecular docking

Candidate compounds were initially screened from the TargetMol T001 and D001 libraries using a structure-based virtual screening strategy, and representative small molecules with high predicted affinity for the target protein were selected for further docking analysis. The molecular docking procedure was conducted with CB-Dock2 (http://cao.labshare.cn/cb-dock2/), a web-based tool built on the AutoDock Vina algorithm. Three-dimensional structures of the target protein were retrieved from the PDB in PDB format, and the selected small molecules were converted into MOL2 format. CB-Dock2 automatically detected potential binding pockets on the protein surface and docked each ligand into the predicted cavities. For each docking run, multiple binding conformations were generated, and the corresponding Vina scores were calculated to estimate binding affinity. The docking results, including the lowest-energy conformations and predicted interaction modes, were further visualized and analyzed within the CB-Dock2 platform.

### 4.16 Statistical methods

Data were statistically analyzed using GraphPad Prism (8.4.3) and R software (4.4.1). Normally distributed variables were analyzed using the t-test. Non-normally distributed variables were analyzed using the Wilcoxon rank sum test. p<0.05 was considered statistically significant.

## Data Availability

The original contributions presented in the study are included in the article/**Supplementary Material**. Further inquiries can be directed to the corresponding authors.

## Supplementary material

Supplementary Figure S1 **(A)** Venn diagram illustrating the overlap of hub genes identified by three supervised machine learning approaches. **(B, C)** Mutation landscape of the low- and high-risk groups. **(D, E)** Kaplan–Meier plots validating the predictive power of prognostic models with the GSE26939 dataset. **(F, G)** Kaplan–Meier plots validating the predictive power of prognostic models with the GSE3141 dataset. **(H)** Time-dependent AUC values at 1 year, 3 years, and 5 years were compared across our HALARG model and published prognostic signatures reported in the indicated studies. The HALARG model is highlighted in red, the hypoxia-based model in green, the lactylation-based model in orange, and the other published signatures in blue. **(I)** Network of cell–cell interactions among immune cells; Orange lines indicate positive correlations, green lines indicate negative correlations, and line thickness represents correlation strength. **(J)** Proportions of responders and non-responders to immune checkpoint blockade in the HALARG-high and HALARG-low groups in the GSE126044 cohort. Comparison of HALARG scores between responders and non-responders to immune checkpoint blockade in the GSE126044 cohort. **(K)** Proportions of responders and non-responders to immune checkpoint blockade in the HALARG-high and HALARG-low groups in the GSE135222 cohort. Comparison of HALARG scores between responders and non-responders to immune checkpoint blockade in the GSE135222 cohort.

Supplementary Figure S2 **(A)** UMAP plot showing the distribution of high- and low-HALARG-score groups across scRNA-seq samples. **(B)** Bar plot comparing the overall interaction strength between high- and low- HALARG-score groups. **(C)** UMAP projections colored by the lactylation-related and hypoxia-related module scores (Lacty_score and Hypo_score), respectively. **(D)** Representative UMAP feature plots showing the expression distributions of hypoxia- and lactylation-associated genes, including SLC2A1, LDHA, PABPC1, and ENO1, in the integrated scRNA-seq dataset. **(E, F)** Heatmaps displaying incoming and outgoing signaling patterns of the high- and low-HALARG-score groups.

Supplementary Figure S3 **(A)** Violin plot showing the distribution of HALARG scores across major cell lineages in the integrated scRNA-seq dataset; statistical comparisons between cell types are indicated. **(B)** tSNE of T-cell subtypes, including CD4^+^ T naïve, CD4^+^ T, CD4^+^ Treg, CD8^+^ T effector, and γδ T cells. **(C)** Stacked bar plot comparing the composition of T-cell subtypes between high- and low-HALARG-score groups. **(D)** Violin plot comparing the T-cell exhaustion score between high- and low-HALARG-score groups. **(E)** tSNE embedding of myeloid subtypes, including cDC1, cDC2, cDC3, macrophages, and monocytes. **(F)** Stacked bar plot comparing the composition of myeloid subtypes between high- and low-HALARG-score groups. **(G)** Violin plot comparing the M1 polarization score between high- and low-HALARG-score groups. **(H)** Violin plot comparing the M2 polarization score between high- and low-HALARG-score groups.

Supplementary Figure S4 Molecular docking of drugs. Molecular docking diagrams of PABPC1 with the top two compounds, castanospermine **(A)** and arimoclomol maleate **(B)** showing the strongest binding affinities.

## References

[1] Thai AA Solomon BJ Sequist LV Gainor JF Heist RS . Lung cancer. Lancet (London England). (2021) 398:535–54. doi: 10.1016/s0140-6736(21)00312-3. PMID: 34273294

[2] Nooreldeen R Bach H . Current and future development in lung cancer diagnosis. Int J Mol Sci. (2021) 22(16). doi: 10.3390/ijms22168661. PMID: 34445366 PMC8395394

[3] Liu L Soler J Reckamp KL Sankar K . Emerging targets in non-small cell lung cancer. Int J Mol Sci. (2024) 25. doi: 10.3390/ijms251810046. PMID: 39337530 PMC11432526

[4] Wang WW Liu Y He Z Li L Liu S Jiang M . Breakthrough of solid tumor treatment: CAR-NK immunotherapy. Cell Death Discov. (2024) 10:40. doi: 10.1038/s41420-024-01815-9. PMID: 38245520 PMC10799930

[5] Meng W Hao Y He C Li L Zhu G . Exosome-orchestrated hypoxic tumor microenvironment. Mol Cancer. (2019) 18:57. doi: 10.1186/s12943-019-0982-6. PMID: 30925935 PMC6441221

[6] Jing X Yang F Shao C Wei K Xie M Shen H . Role of hypoxia in cancer therapy by regulating the tumor microenvironment. Mol Cancer. (2019) 18:157. doi: 10.1186/s12943-019-1089-9. PMID: 31711497 PMC6844052

[7] Wigerup C Påhlman S Bexell D . Therapeutic targeting of hypoxia and hypoxia-inducible factors in cancer. Pharmacol Ther. (2016) 164:152–69. doi: 10.1016/j.pharmthera.2016.04.009. PMID: 27139518

[8] Chen Y Yan H Yan L Wang X Che X Hou K . Hypoxia-induced ALDH3A1 promotes the proliferation of non-small-cell lung cancer by regulating energy metabolism reprogramming. Cell Death Dis. (2023) 14:617. doi: 10.1038/s41419-023-06142-y. PMID: 37730658 PMC10511739

[9] Xiong L He X Wang L Dai P Zhao J Zhou X . Hypoxia-associated prognostic markers and competing endogenous RNA coexpression networks in lung adenocarcinoma. Sci Rep. (2022) 12:21340. doi: 10.1038/s41598-022-25745-7. PMID: 36494419 PMC9734750

[10] Wu Q You L Nepovimova E Heger Z Wu W Kuca K . Hypoxia-inducible factors: master regulators of hypoxic tumor immune escape. J Hematol Oncol. (2022) 15:77. doi: 10.1186/s13045-022-01292-6. PMID: 35659268 PMC9166526

[11] Lv X Lv Y Dai X . Lactate, histone lactylation and cancer hallmarks. Expert Rev Mol Med. (2023) 25:e7. doi: 10.1017/erm.2022.42. PMID: 36621008

[12] Lin J Liu G Chen L Kwok HF Lin Y . Targeting lactate-related cell cycle activities for cancer therapy. Semin Cancer Biol. (2022) 86:1231–43. doi: 10.1016/j.semcancer.2022.10.009. PMID: 36328311

[13] Li H Sun L Gao P Hu H . Lactylation in cancer: Current understanding and challenges. Cancer Cell. (2024) 42:1803–7. doi: 10.1016/j.ccell.2024.09.006. PMID: 39393355

[14] Llibre A Kucuk S Gope A Certo M Mauro C . Lactate: A key regulator of the immune response. Immunity. (2025) 58:535–54. doi: 10.1016/j.immuni.2025.02.008. PMID: 40073846

[15] Faubert B Li KY Cai L Hensley CT Kim J Zacharias LG . Lactate metabolism in human lung tumors. Cell. (2017) 171:358–71.e9. doi: 10.1016/j.cell.2017.09.019. PMID: 28985563 PMC5684706

[16] Qu J Li P Sun Z . Histone lactylation regulates cancer progression by reshaping the tumor microenvironment. Front Immunol. (2023) 14:1284344. doi: 10.3389/fimmu.2023.1284344. PMID: 37965331 PMC10641494

[17] Zhang C Zhou L Zhang M Du Y Li C Ren H . H3K18 lactylation potentiates immune escape of non-small cell lung cancer. Cancer Res. (2024) 84:3589–601. doi: 10.1158/0008-5472.can-23-3513. PMID: 39137401

[18] Wang ZH Peng WB Zhang P Yang XP Zhou Q . Lactate in the tumour microenvironment: From immune modulation to therapy. EBioMedicine. (2021) 73:103627. doi: 10.1016/j.ebiom.2021.103627. PMID: 34656878 PMC8524104

[19] Babl N Decking SM Voll F Althammer M Sala-Hojman A Ferretti R . MCT4 blockade increases the efficacy of immune checkpoint blockade. J Immunother Cancer. (2023) 11(10). doi: 10.1136/jitc-2023-007349. PMID: 37880183 PMC10603342

[20] Romero Y Aquino-Gálvez A . Hypoxia in cancer and fibrosis: Part of the problem and part of the solution. Int J Mol Sci. (2021) 22(15). doi: 10.3390/ijms22158335. PMID: 34361103 PMC8348404

[21] Huang Y Chen Z Lu T Bi G Li M Liang J . HIF-1α switches the functionality of TGF-β signaling via changing the partners of smads to drive glucose metabolic reprogramming in non-small cell lung cancer. J Exp Clin Cancer Research: CR. (2021) 40:398. doi: 10.1891/9780826145987.0003. PMID: 34930376 PMC8690885

[22] Chen L Huang L Gu Y Cang W Sun P Xiang Y . Lactate-lactylation hands between metabolic reprogramming and immunosuppression. Int J Mol Sci. (2022) 23(19). doi: 10.3390/ijms231911943. PMID: 36233246 PMC9569569

[23] Feng J Dai W Mao Y Wu L Li J Chen K . Simvastatin re-sensitizes hepatocellular carcinoma cells to sorafenib by inhibiting HIF-1α/PPAR-γ/PKM2-mediated glycolysis. J Exp Clin Cancer Research: CR. (2020) 39:24. doi: 10.1186/s13046-020-1528-x. PMID: 32000827 PMC6993409

[24] Yarla NS Bishayee A Sethi G Reddanna P Kalle AM Dhananjaya BL . Targeting arachidonic acid pathway by natural products for cancer prevention and therapy. Semin Cancer Biol. (2016) 40-41:48–81. doi: 10.1016/j.semcancer.2016.02.001. PMID: 26853158

[25] Tredicine M Mucci M Recchiuti A Mattoscio D . Immunoregulatory mechanisms of the arachidonic acid pathway in cancer. FEBS Lett. (2025) 599:927–51. doi: 10.1002/1873-3468.70013. PMID: 39973474 PMC11995684

[26] Gao Q Yang L Lu M Jin R Ye H Ma T . The artificial intelligence and machine learning in lung cancer immunotherapy. J Hematol Oncol. (2023) 16:55. doi: 10.1186/s13045-023-01456-y. PMID: 37226190 PMC10207827

[27] De Visser KE Joyce JA . The evolving tumor microenvironment: From cancer initiation to metastatic outgrowth. Cancer Cell. (2023) 41:374–403. doi: 10.1016/j.ccell.2023.02.016. PMID: 36917948

[28] Gonçalves AC Richiardone E Jorge J Polónia B Xavier CPR Salaroglio IC . Impact of cancer metabolism on therapy resistance - Clinical implications. Drug Resistance Updates: Rev Commentaries Antimicrobial Anticancer Chemotherapy. (2021) 59:100797. 10.1016/j.drup.2021.100797 34955385

[29] Vignali PDA Depeaux K Watson MJ Ye C Ford BR Lontos K . Hypoxia drives CD39-dependent suppressor function in exhausted T cells to limit antitumor immunity. Nat Immunol. (2023) 24:267–79. doi: 10.1038/s41590-022-01379-9. PMID: 36543958 PMC10402660

[30] Scharping NE Rivadeneira DB Menk AV Vignali PDA Ford BR Rittenhouse NL . Mitochondrial stress induced by continuous stimulation under hypoxia rapidly drives T cell exhaustion. Nat Immunol. (2021) 22:205–15. doi: 10.1038/s41590-020-00834-9. PMID: 33398183 PMC7971090

[31] Qi Y Wang M Jiang Q . PABPC1--mRNA stability, protein translation and tumorigenesis. Front Oncol. (2022) 12:1025291. doi: 10.3389/fonc.2022.1025291. PMID: 36531055 PMC9753129

[32] Lin M Hu L Shen S Liu J Liu Y Xu Y . Atherosclerosis-related biomarker PABPC1 predicts pan-cancer events. Stroke Vasc Neurol. (2024) 9:108–25. doi: 10.1136/svn-2022-002246. PMID: 37311641 PMC11103157

[33] Li C Han T Li Q Zhang M Guo R Yang Y . MKRN3-mediated ubiquitination of Poly(A)-binding proteins modulates the stability and translation of GNRH1 mRNA in mammalian puberty. Nucleic Acids Res. (2021) 49:3796–813. doi: 10.1093/nar/gkab155. PMID: 33744966 PMC8053111

[34] Gao M Wang M Zhou S Hou J He W Shu Y . Machine learning-based prognostic model of lactylation-related genes for predicting prognosis and immune infiltration in patients with lung adenocarcinoma. Cancer Cell Int. (2024) 24:400. doi: 10.1186/s12935-024-03592-y. PMID: 39696439 PMC11656871

[35] Warde-Farley D Donaldson SL Comes O Zuberi K Badrawi R Chao P . The GeneMANIA prediction server: biological network integration for gene prioritization and predicting gene function. Nucleic Acids Res. (2010) 38:W214–20. doi: 10.1093/nar/gkq537. PMID: 20576703 PMC2896186

[36] Mariathasan S Turley SJ Nickles D Castiglioni A Yuen K Wang Y . TGFβ attenuates tumour response to PD-L1 blockade by contributing to exclusion of T cells. Nature. (2018) 554:544–8. doi: 10.1038/nature25501. PMID: 29443960 PMC6028240

[37] Schubert M Klinger B KlüNemann M Sieber A Uhlitz F Sauer S . Perturbation-response genes reveal signaling footprints in cancer gene expression. Nat Commun. (2018) 9:20. doi: 10.1038/s41467-017-02391-6. PMID: 29295995 PMC5750219

[38] Lawson KA Sousa CM Zhang X Kim E Akthar R Caumanns JJ . Functional genomic landscape of cancer-intrinsic evasion of killing by T cells. Nature. (2020) 586:120–6. doi: 10.1038/s41586-020-2746-2. PMID: 32968282 PMC9014559

[39] Wang Z Wang Y Chang M Wang Y Liu P Wu J . Single-cell transcriptomic analyses provide insights into the cellular origins and drivers of brain metastasis from lung adenocarcinoma. Neuro-Oncology. (2023) 25:1262–74. doi: 10.1093/neuonc/noad017. PMID: 36656750 PMC10326480

