# Supplementary material for: A machine learning-derived hypoxia- and lactylation-associated gene signature for prognostic stratification and immune landscape characterization in lung adenocarcinoma
Source: Front Immunol. 2026 May 1;17:1720885. doi: 10.3389/fimmu.2026.1720885 (PMC13176175; doi:10.3389/fimmu.2026.1720885)
Supplement: Supplementary Figure S1 — (A) Venn diagram illustrating the overlap of hub genes identified by three supervised machine learning approaches. (B, C) Mutation landscape of the low- and high-risk groups. (D, E) Kaplan–Meier plots validating the predictive power of prognostic models with the GSE26939 dataset. (F, G) Kaplan–Meier plots validating the predictive power of prognostic models with the GSE3141 dataset. (H) Time-dependent AUC values at 1 year, 3 years, and 5 years were compared across our HALARG model and published prognostic signatures reported in the indicated studies. The HALARG model is highlighted in red, the hypoxia-based model in green, the lactylation-based model in orange, and the other published signatures in blue. (I) Network of cell–cell interactions among immune cells; Orange lines indicate positive correlations, green lines indicate negative correlations, and line thickness represents correlation strength. (J) Proportions of responders and non-responders to immune checkpoint blockade in the HALARG-high and HALARG-low groups in the GSE126044 cohort. Comparison of HALARG scores between responders and non-responders to immune checkpoint blockade in the GSE126044 cohort. (K) Proportions of responders and non-responders to immune checkpoint blockade in the HALARG-high and HALARG-low groups in the GSE135222 cohort. Comparison of HALARG scores between responders and non-responders to immune checkpoint blockade in the GSE135222 cohort. [file SupplementaryFile1.pdf]

Fig. s1

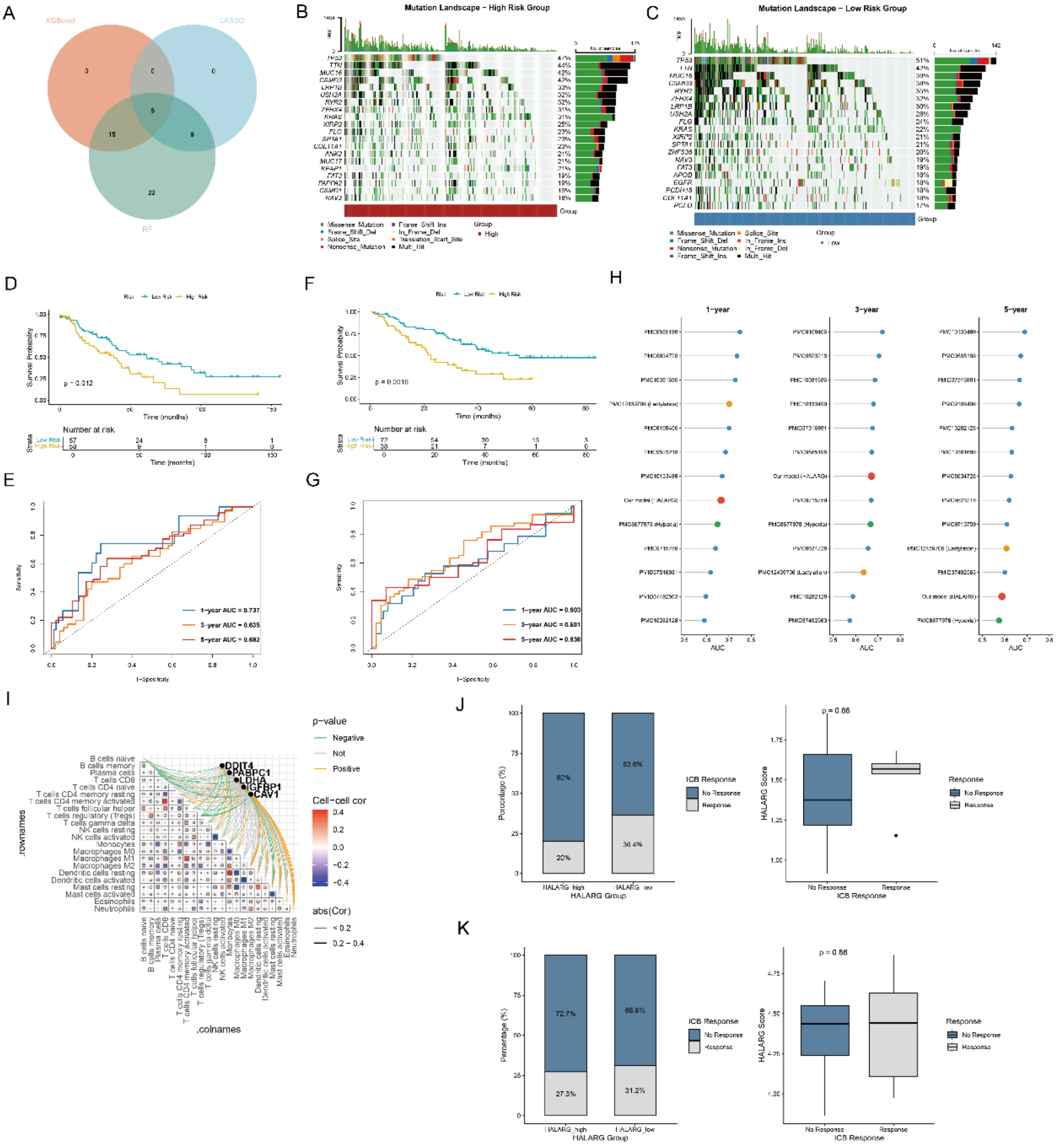

Supplementary Figure 1. (A) Venn diagram illustrating the overlap of hub genes identified by three supervised machine learning approaches. (B, C) Mutation landscape of the low- and high-risk groups. (D, E) Kaplan–Meier plots validating the predictive power of prognostic models with the GSE26939 dataset. (F, G) Kaplan–Meier plots validating the predictive power of prognostic models with the GSE3141 dataset. (H) Time-dependent AUC values at 1 year, 3 years, and 5 years were compared across our HALARG model and published prognostic signatures reported in the indicated studies. The HALARG model is highlighted in red, the hypoxia-based model in green, the lactylation-based model in orange, and the other published signatures in blue. (I) Network of cell–cell interactions among immune cells; Orange lines indicate positive correlations, green lines indicate negative correlations, and line thickness represents correlation strength. (J) Proportions of responders and non-responders to immune checkpoint blockade in the HALARG-high and HALARG-low groups in the GSE126044 cohort. Comparison of HALARG scores between responders and non-responders to immune checkpoint blockade in the GSE126044 cohort. (K) Proportions of responders and non-responders to immune checkpoint blockade in the HALARG-high and HALARG-low groups in the GSE135222

cohort. Comparison of HALARG scores between responders and non-responders to immune checkpoint blockade in the GSE135222 cohort.

Fig. s2

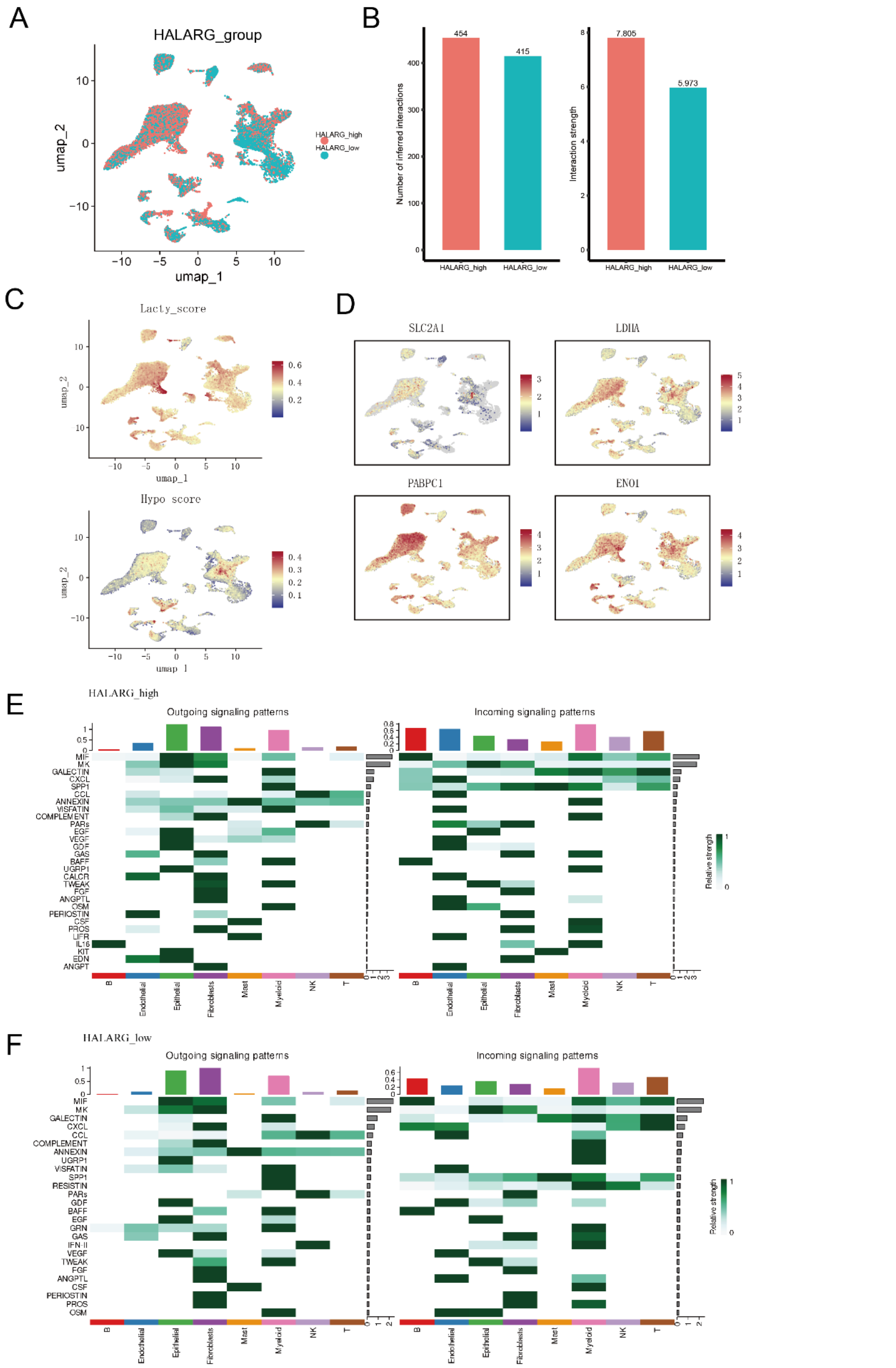

Supplementary Figure 2 (A) UMAP plot showing the distribution of high- and low-HALARG-score groups across scRNA-seq samples. (B) Bar plot comparing the overall interaction strength between

high- and low- HALARG-score groups. (C) UMAP projections colored by the lactylation-related and hypoxia-related module scores (Lacty_score and Hypo_score), respectively. (D) Representative UMAP feature plots showing the expression distributions of hypoxia- and lactylation-associated genes, including SLC2A1, LDHA, PABPC1, and ENO1, in the integrated scRNA-seq dataset. (E, F) Heatmaps displaying incoming and outgoing signaling patterns of the high- and low-HALARG-score groups.

Fig. s3

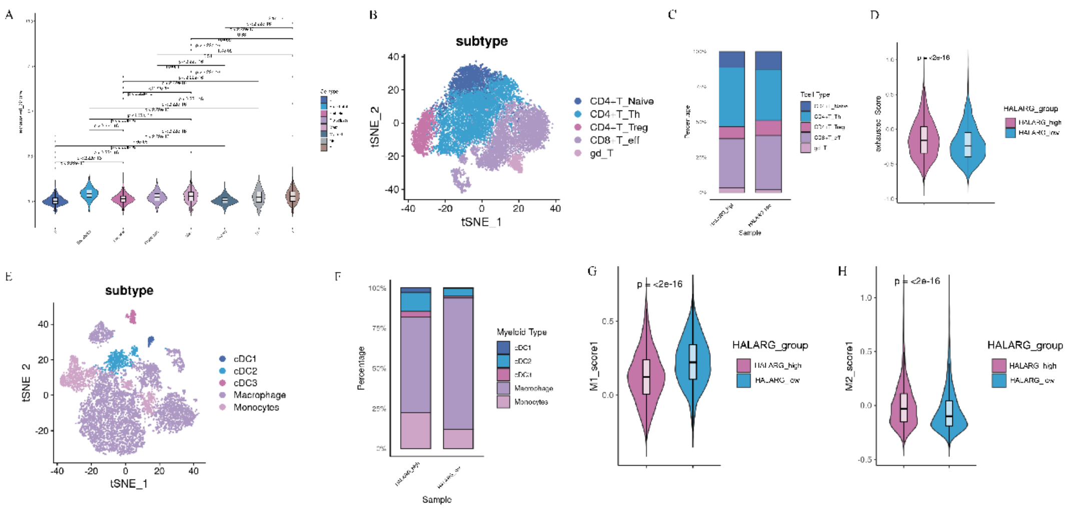

Supplementary Figure 3 (A) Violin plot showing the distribution of HALARG scores across major cell lineages in the integrated scRNA-seq dataset; statistical comparisons between cell types are indicated. (B) tSNE of T-cell subtypes, including CD4^{+} T naïve, CD4^{+} T, CD4^{+} Treg, CD8^{+} T effector, and γδ T cells. (C) Stacked bar plot comparing the composition of T-cell subtypes between high- and low-HALARG-score groups. (D) Violin plot comparing the T-cell exhaustion score between high- and low-HALARG-score groups. (E) tSNE embedding of myeloid subtypes, including cDC1, cDC2, cDC3, macrophages, and monocytes. (F) Stacked bar plot comparing the composition of myeloid subtypes between high- and low-HALARG-score groups. (G) Violin plot comparing the M1 polarization score between high- and low-HALARG-score groups. (H) Violin plot comparing the M2 polarization score between high- and low-HALARG-score groups.

Fig. s4

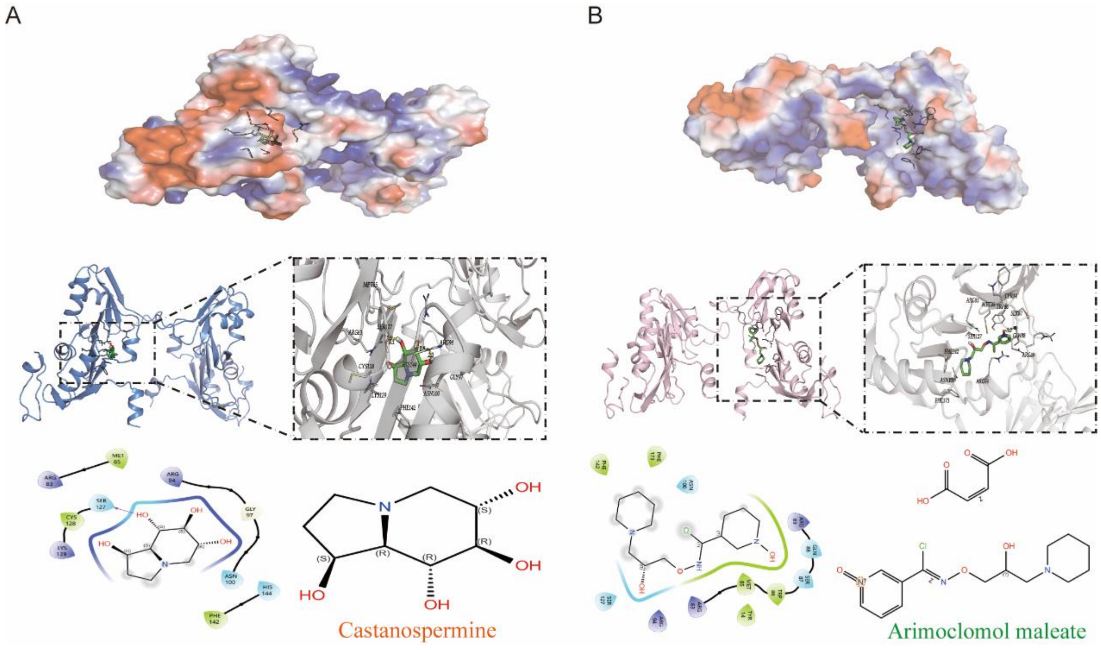

Supplementary Figure 4 Molecular docking of drugs. Molecular docking diagrams of PABPC1 with the top two compounds, castanospermine (A) and arimoclomol maleate (B) showing the strongest binding affinities.
